# In-silico personalized protein–protein interaction networks prioritize candidate compounds for glioblastoma

**DOI:** 10.1371/journal.pcbi.1014785

**Published:** 2026-09-22

**Authors:** Nicoleta Siminea, Victor-Bogdan Popescu, Mihaela Păun, Ion Petre, Andrei Păun

**Affiliations:** 1 Faculty of Mathematics and Computer Science, University of Bucharest, Bucharest, Romania; 2 Bioinformatics, National Institute of Research and Development for Biological Sciences, Bucharest, Romania; 3 Revvity Inc., Turku, Finland; 4 Faculty of Business and Administration, University of Bucharest, Bucharest, Romania; 5 Department of Mathematics and Statistics, University of Turku, Turku, Finland; 6 Research Institute for Artificial Intelligence “Mihai Drăgănescu”, Romanian Academy, Bucharest, Romania; University of Pisa: Universita degli Studi di Pisa, ITALY

## Abstract

The incidence of cancer continues to rise globally, with many tumor types remaining difficult to treat effectively. While existing drugs can alleviate symptoms or slow disease progression, their efficacy varies considerably between patients. To explore this challenge, we present a computational approach for identifying patient-specific candidate compounds. Our approach was developed for glioblastoma, but it can be applied to other types of cancer. Our study involves identifying possible disease-relevant proteins specific to each patient that underlie the generated networks, together with drug targets and proteins associated with cancer dependency. These networks are analyzed using centrality measures and Louvain community detection method. Then, further investigating the individual networks, network controllability analysis is applied to identify key regulatory targets within the network. These targets are filtered and ranked to prioritize candidate drugs for individualized treatment. By comparing results across patients and against networks based on generic data, we show substantial differences between patient-specific and generic network representations. Additionally, we track changes in patient-specific networks over time in five glioblastoma cases, exploring how molecular differences between primary and recurrent tumors may influence network structure and computational drug prioritization.

## Author summary

Our study was motivated by the poor prognosis of glioblastoma and by the possibility that compounds not identified across all patients may still be relevant in specific molecular contexts. Therefore, our goal was to identify candidate compounds at the individual patient level using a computational network-based approach. To achieve this, we constructed a network for each sample by integrating proteins encoded by differentially expressed genes and mutated genes, along with proteins known to influence cancer progression and the targets of therapeutic drugs. These networks were based on protein-protein interactions, as they are well suited for capturing network-level effects. The study was conducted from a broad perspective to a detailed analysis. We identified hubs across all the networks, then divided the samples in communities and characterized them to understand the different ways the disease might progress. Next, we applied network controllability on each network as our main analytical method. Our in silico results suggest substantial differences between individual patient networks and a generalized network representation, indicating that patient-specific molecular context can influence computational drug prioritization. The identified compounds should be regarded as computationally prioritized candidates requiring further biological and clinical validation.

## Introduction

Drugs developed for specific diseases may not always be effective for every patient and the response rates can vary significantly depending on the disease and the treatment applied, and one contributing factor is individual genetic variability [1]. Increasingly, clinicians are incorporating genetic and molecular data into the diagnostic and treatment processes [2].

The concept of personalized medicine (selecting the optimal treatment based on a patient’s genetic profile) is increasingly recognized [3]. For instance, cetuximab is recommended only in patients with wild-type KRAS in colorectal cancer [4], and mutations in BRCA1 and BRCA2 are now well-established risk factors in breast cancer [5], enabling preventive strategies.

Numerous authors have highlighted the potential benefits of personalized treatment options for various diseases, such as non-small cell lung cancer [6] and multiple sclerosis [7]. There have also been attempts to stratify treatments in order to achieve better outcomes in conditions like colorectal cancer [8] and breast cancer [9], among others.

In parallel, the emergence of artificial intelligence (AI) has further advanced this paradigm. AI-based models are increasingly capable of uncovering complex disease patterns and recommending personalized interventions. For example, DeepSurv [10] is a deep neural network based on the Cox proportional hazards model that provides personalized survival predictions, while DeepDRK [11] integrates multi-omics data to identify personalized drug - target interactions in cancer, whereas DeepDR [12] is a deep-learning library for drug-response prediction. However, although deep learning models often achieve strong predictive performance, they generally lack interpretability. Moreover, compared with such models, protein-protein interaction (PPI) networks are particularly advantageous when sample sizes are limited. Importantly, PPI networks offer a complementary perspective for personalized therapy, as PPI-based analyses yield biologically interpretable results and provide possible mechanistic insights into how candidate drugs may modulate disease-related pathways. Several network-based methods can be applied to PPI networks; one such approach is network controllability [13]. The idea of identifying personalized therapeutic targets through network controllability is not new. For example, Guo et al. [14] used genetic data to infer disease drivers from co-expression networks. Our approach differs in two important ways. First, we work with protein-protein interaction (PPI) networks rather than co-expression networks. Second, our goal is not to identify disease drivers; instead, we aim to determine potential key nodes within the network that enable us to reach candidate proteins encoded by genes known to promote cancer progression-proteins that appear within patient-specific networks. In this sense, our method is complementary to that of [14].

It is well-established that diseases are typically not caused by a single gene; rather, their occurrence usually involves multiple genes that the body cannot regulate effectively [15]. Moreover, the field of genetics has advanced significantly, yielding extensive datasets that can be analyzed to identify candidate treatment options [16]. Mutations can be readily detected through experimental results, and there are numerous algorithms and software packages available that facilitate the identification of overexpressed and underexpressed genes efficiently [17,18]. Various strategies exist for managing large volumes of genetic data [19]. Networks can be constructed using a diversity of methods, depending on the application [20]. For instance, a network could be constructed by concentrating solely on the interactions between proteins encoded by genes that exhibit differential expression in the patient under examination [21]. Alternatively, the network could encompass the largest component anchored on disease proteins, but also including non-disease connector proteins [22]. The interactions considered could be either directed [23] or undirected [24].

This study utilized biological networks encompassing all points of interest, which include proteins specific to the sample being analyzed, proteins crucial to the progression of the disease, and proteins that are potential targets for therapeutic interventions. Therefore, the network building strategy was selected based on the requirements of the method intended for use in the subsequent step. All decisions made during the network creation process, as well as those made during the analysis of results from the control analyses, are described below. The hypothetical pharmacological agents identified for each individual case were systematically compared with those identified by other in silico method, as well as with the outcomes obtained from a generic network comprised solely of proteins identified as being overexpressed or underexpressed in cancer. This comparison illustrates that patient-specific and generic network representations can yield substantially different drug prioritizations.

All analyses were done on glioblastoma, a disease with a 5% survival rate at 5 years [25] even with optimal treatment options. The choice of this disease was made due to its poor prognosis and the general ineffectiveness of standard treatments. It is important to note that if a disease can be treated successfully with a treatment administered regardless of patient characteristics, extracting genetic data and performing these analyses would be unnecessary.

Treatment for glioblastoma encompasses not only pharmacological options but also a comprehensive array of interventions tailored based on criteria including age, tumor spread, and the presence of comorbidities. The standard approach involves surgical resection of the tumor when deemed beneficial, performed in a manner that minimizes the impact on brain functions. Depending on these criteria, adjuvant radiation therapy may be administered. Temozolomide is typically considered the first line pharmacologic treatment [26]. For recurrent or progressive tumors, other options such as bevacizumab or nitrosoureas (including lomustine, carmustine, and fotemustine) may be utilized [27]. Furthermore, patients may receive palliative care involving antiepileptics, corticosteroids, or other medications as indicated by their specific clinical scenario [28].

The concept of personalizing drug therapy for glioblastoma based on genetic and molecular data has been previously explored through various approaches. One of the early efforts was by [29], who tailored treatments according to glioblastoma subtypes – proneural, neural, classical, and mesenchymal. Another notable study by [30] developed a three-dimensional model of the patient’s infiltrative glioblastoma microenvironment to facilitate more precise therapeutic selection. A retrospective analysis conducted by [31] involved molecular profiling of 50 brain tumors and identified individualized treatment options for 35 of the cases. More recently [32], performed a comparative study involving 41 glioblastoma patients, where 18 received therapy based on sequencing data, while the remaining 23 underwent standard treatment. Actionable targets were identified in 31 of the 41 patients, with favorable outcomes reported for those who received personalized therapies.

**Fig 1:**
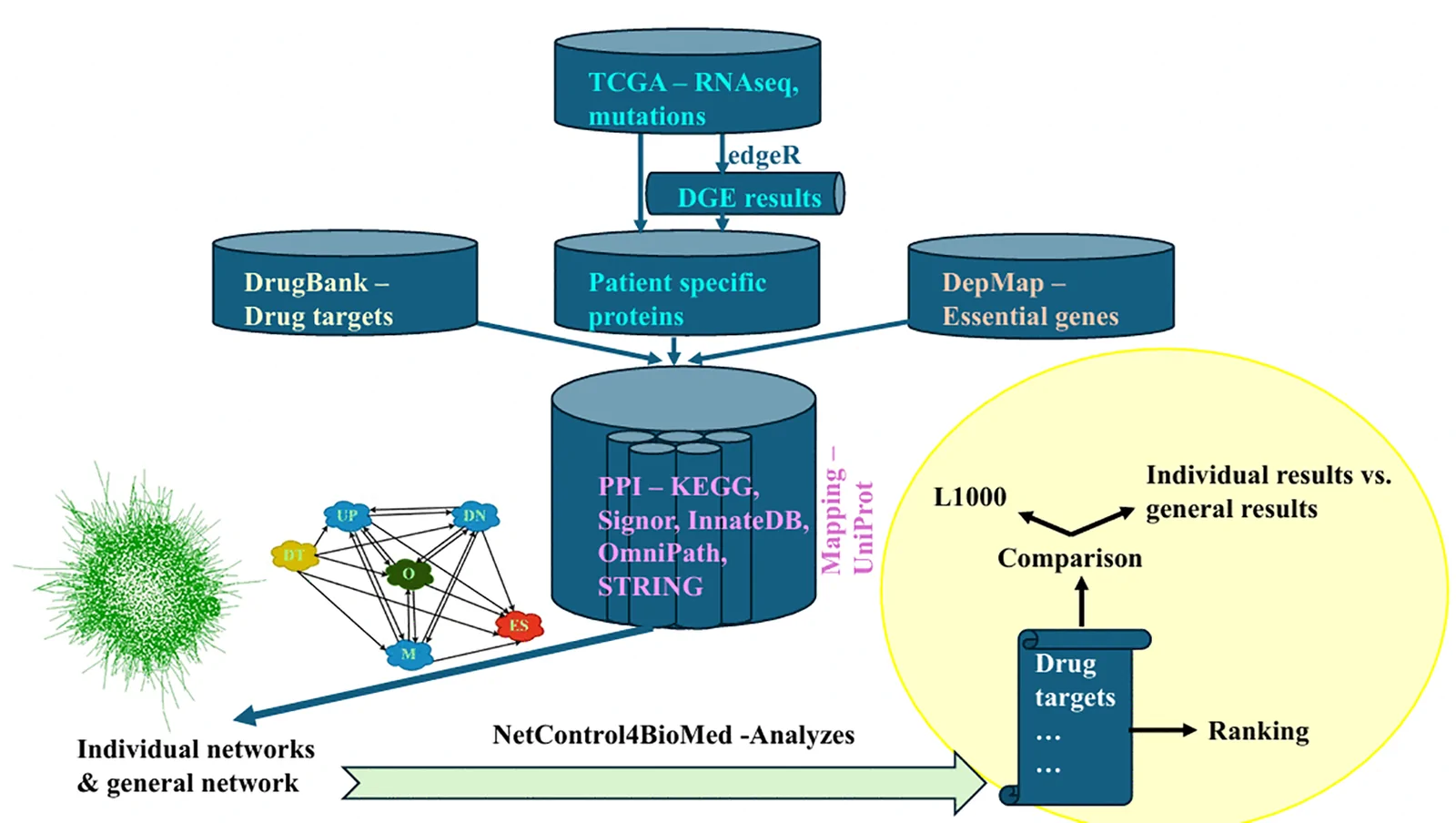
The study workflow. We analyzed RNA-seq data and corresponding mutation profiles for the same cases from the TCGA-GBM study. For each case, we identified differentially expressed genes and combined them with the associated mutations to define a case-specific gene set. We then integrated drug targets from DrugBank and cancer dependency proteins to construct interaction networks using data from KEGG, SIGNOR, InnateDB, OmniPath, and STRING. Both individual case networks and a global network were generated and systematically characterized. Subsequently, we performed controllability analyses, comparing the results with those obtained from the L1000 dataset, as well as across individual cases and the global network.

In this study, we constructed a directed protein-protein interaction network for each patient, incorporating proteins encoded by up-regulated and down-regulated genes, as well as those encoded by mutant genes. We characterized the obtained networks using centralities as well as communities. Next, we performed network controllability analysis to identify key regulatory proteins – those proteins capable of controlling the majority of proteins critical for tumor survival or proliferation. Following this, we searched for drugs targeting the identified key regulators. The candidate drugs were selected from a broad range of anticancer and immunomodulatory agents used in various cancer types, not exclusively glioblastoma. The study workflow is presented in Fig 1.

## Results

### Differentially expressed genes

We extracted RNA-seq data from The Cancer Genome Atlas (TCGA) [33], the GBM project (Glioblastoma Multiforme Project). Our data includes 137 primary glioblastoma tumor samples, 13 recurrent glioblastoma tumor samples, and 4 tumor-free control samples. Five patients contributed both primary and recurrent tumor samples; the remaining tumor samples corresponded to distinct patients. Additional details on the dataset source and identifiers are provided in Materials and Methods. We performed two differential expression analyses: one comparing primary tumor samples to control tissue, and another comparing recurrent tumor samples to control tissue. After identifying differentially expressed genes at the cohort level, we considered a gene as up-regulated/down-regulated at the individual network, applying the threshold presented in detail in the Materials and Methods.

The number of up-regulated genes per primary tumor sample ranged from 150 to 896, while down-regulated genes ranged from 29 to 1061. For recurrent cases, the up-regulated genes were between 235 and 778, and the down-regulated genes were between 258 and 461. Fewer differentially expressed genes were observed in the recurrent cohort; given the small number of recurrent samples, this difference should be interpreted cautiously.

### Mutated genes

For each tumor case analyzed, we extracted single nucleotide variation (SNV) data, which are found within the same TCGA-GBM project, following a case code search. For all tumor cases, we downloaded mutational data and retained only those variants classified as high or moderate impact by the Variant Effect Predictor (VEP). Variants with a high VEP impact are predicted to significantly disrupt protein function, while moderate impact variants may result in less severe but still potentially meaningful alterations to protein activity. Our aim in retrieving this data was to exclude the corresponding proteins from the control paths used in our analyses.

The number of mutations varied widely across samples: eight samples contained no high or moderate impact mutations, while three samples exhibited an exceptionally high mutation burden (almost 1000 mutations and above). The median value of high and moderate VEP classified mutations per sample was under 100. We decided to exclude those with more than 1,000 total mutations, following the approach described by Bailey et al. (2018) [34]. Accordingly, three outlier samples were excluded, resulting in a total of 134 primary samples included in the following analysis.

**Fig 2:**
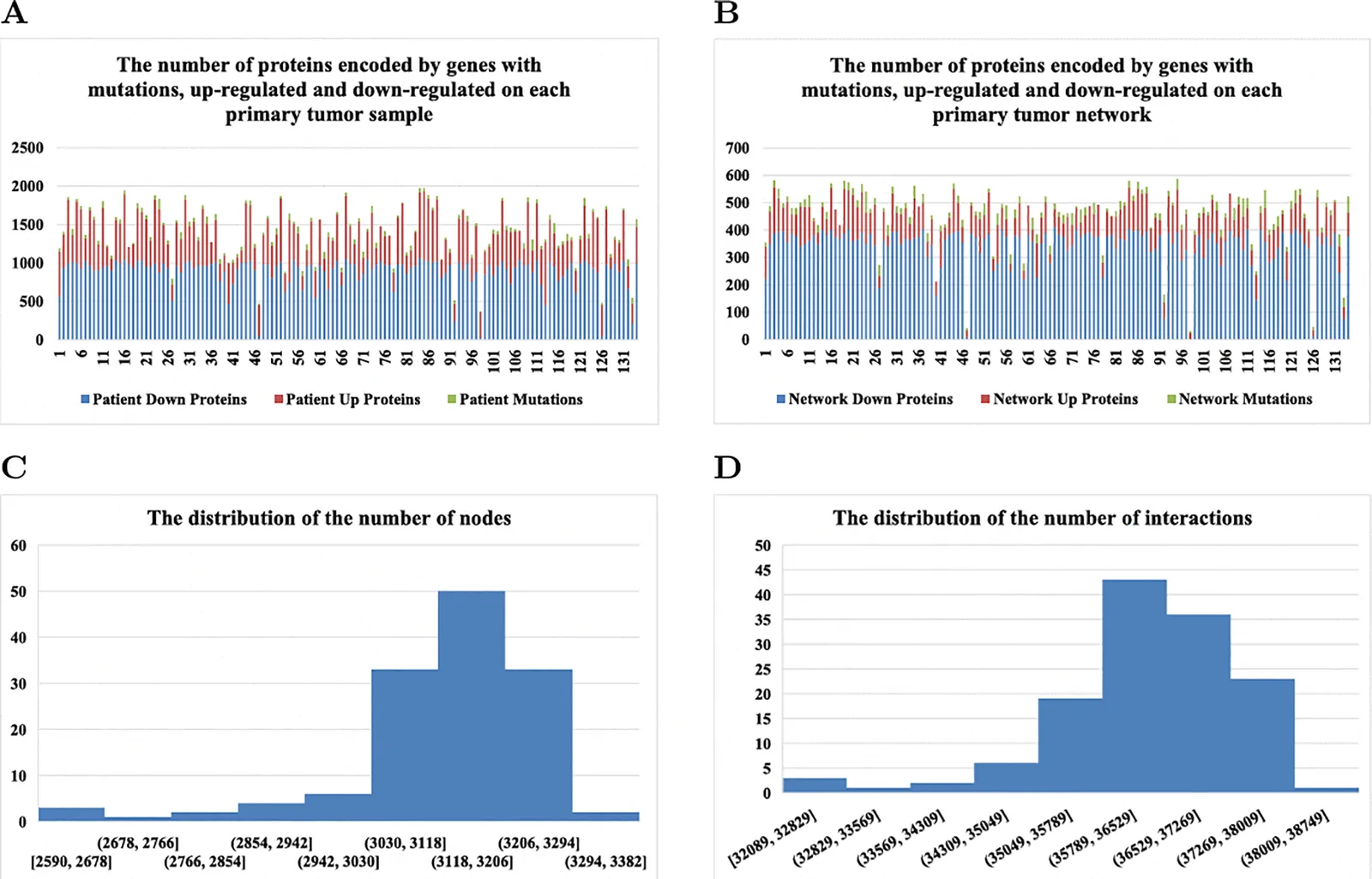
Statistics on primary tumor samples and the associated networks. (A) The number of specific proteins for each sample. (B) The number of specific proteins for each network generated. (C) The range in which the number of nodes per network is found. (D) The range in which the number of interactions on the network is found.

Fig 2A shows the number of proteins encoded by genes with mutations, as well as those that are up-regulated or down-regulated, in each of the remaining primary tumor samples. The data for the recurrent samples is in panel A of S1 Fig.

### Patient-specific interaction networks

We constructed patient-specific directed protein-protein interaction (PPI) networks by integrating both patient-derived molecular data and curated biological data. For each patient, we identified a set of seed proteins that included those encoded by their own differentially expressed genes and their genes carrying high or moderate impact mutations. We supplemented these patient-specific proteins with drug target proteins, as well as proteins encoded by genes of which the progression/existence of the cancer depends (hereinafter referred to as “essential”) because usually they could have a normal expression but their effect could influence the disease. The networks were grown by expanding around these seed proteins using experimentally validated or computationally inferred directed PPIs mined from KEGG [35], OmniPath [36], Signor [37], STRING [38], and InnateDB [39].

By constructing the networks, we eliminated all isolated proteins, which led to eliminating several patient-specific proteins from each sample (Fig 2B and in panel B of S1 Fig). This is likely due to the proteins being too distant from one another within the network. Notably, based on our approach, the number of proteins encoded by down-regulated genes appears to be higher in each sample. These down-regulated proteins also appear to be more interconnected within the networks, as they are represented in greater proportions compared to proteins encoded by up-regulated genes.

In Fig 2C and D, we observe that the average number of nodes in each network is approximately 3,200, while the total number of interactions is around 36,500. More precisely, the generated networks contained between 2,590 and 3,318 nodes for primary cases and between 2845 and 3174 for recurrent cases, which are presented in panels C and D of S1 Fig. The number of interactions varies between 32,089 and 38,173 interactions for primary cases, and between 34529 and 36872 for recurrent cases. In Supplementary Information, the S2 Fig offers a visualization of one of the networks.

The number of drug targets appears to be consistent across samples, with many networks having almost the same number of drug targets, which is about 390. However, based on our in silico study, there are notable differences in the number of specific proteins included in the networks as can be seen from Fig 2.

We performed a topological analysis on each patient-specific network and calculated several graph metrics as described in Materials and Methods section.

In our networks, the network radius was consistently 5 across all networks. In the primary tumor group, the network diameter was typically either 11 or 13, while in the recurrent tumor group, it was consistently 10 (S5 and S6 Figs).

Other global properties, including network density, average shortest path length, and clustering coefficient, showed similar values between primary and recurrent tumor networks, indicating that it might have comparable overall network structure across the two groups (S5 and S6 Figs).

For the computed centralities, we identified the top five hub proteins. The results (S3 Figs for primary tumors and S4 Fig for recurrent tumors) revealed a similar pattern, with the top 5 hubs being the same within both primary and recurrent tumor groups. In addition, across all the centralities, the top hubs that have appeared more than 134 times, together with some of their functions listed in Uniprot [40], are listed in the Table 1.

**Table 1:** The 10 proteins most frequently ranked among the top five hubs across different centrality measures.

Protein	No. of appearances in top 5 hubs	Known functions
SRC	906	controls gene transcription, immune response,
		cell adhesion, cell cycle progression, apoptosis,
		migration, and transformation
TP53	755	induces cell cycle arrest, DNA repair or
		apoptosis; tumor suppressor
UBC	714	in protein degradation, cell cycle regulation,
		DNA damage response
STAT3	485	mediates cellular responses to interleukins;
		role in proliferation, inflammation
CTNNB1	303	role in Wnt signaling and cell adhesion
GSK3B	302	negative regulator in the hormonal control of
		glucose homeostasis
MAPK1	246	controls proliferation, differentiation,
		and survival
EGFR	198	in cell proliferation, survival, migration,
		and differentiation
PTK2	160	in cell migration, adhesion, spreading,
		proliferation, apoptosis, cell cycle progression,
		cytoskeleton, focal adhesions and cell protrusions
CDK1	151	role in cell division (mitosis)
CDK2	151	controls cell cycle

The recurrence of the same hub proteins across patient-specific networks indicates similarity in selected topological features. However, this may partly reflect the central position of these proteins in the underlying interaction resources and should not be interpreted as evidence of common disease mechanisms. Likewise, the similarity of networks obtained after adding intermediate proteins reflects properties of the network reconstruction procedure and does not, by itself, imply convergence of disease progression.

**Fig 3:**
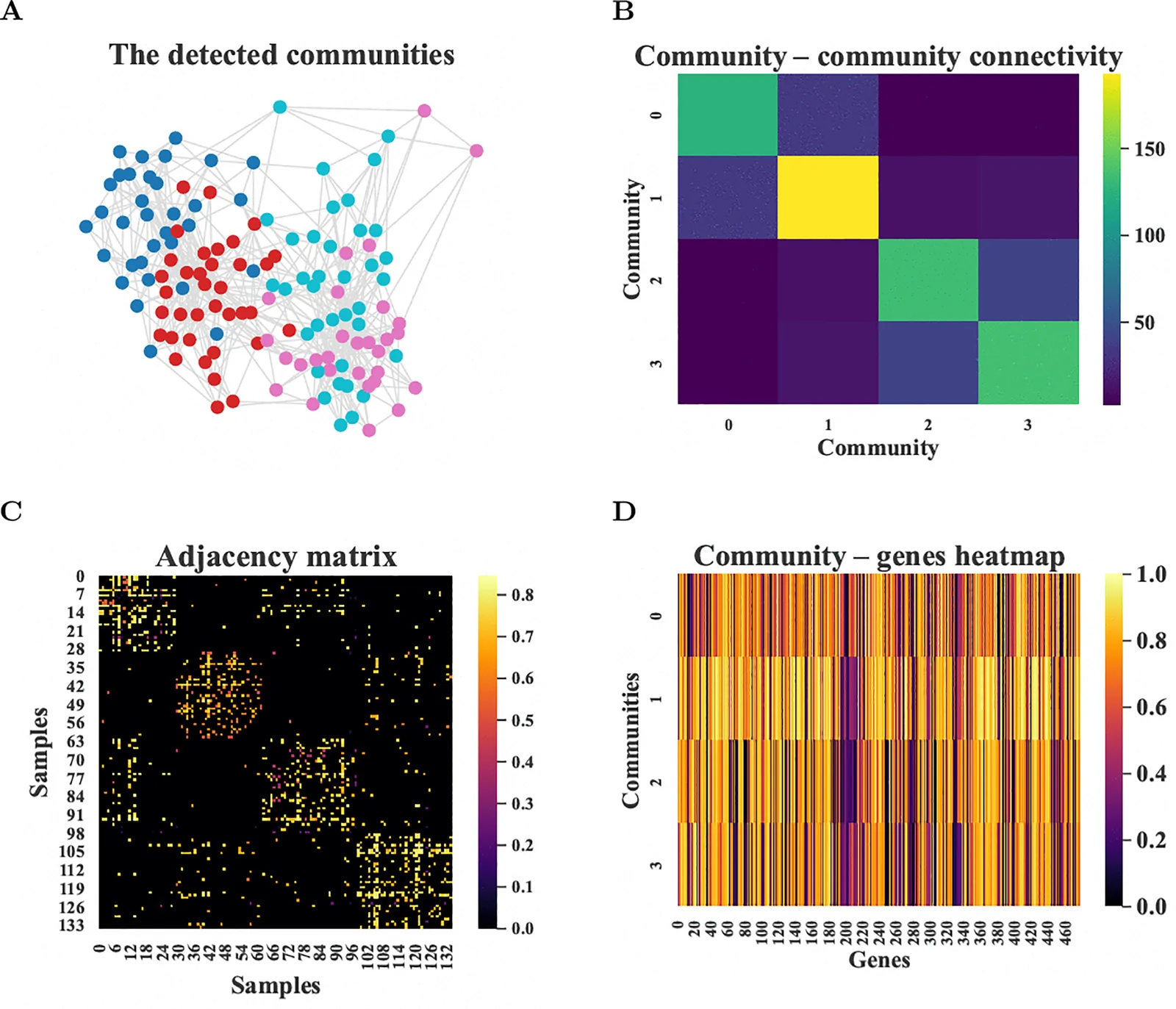
Communities identified on samples network. (A) The network of samples with the communities colored in distinct colors. (B) Heatmap that shows that the number of interactions inside a community is bigger than the number outside. (C) Heatmap that shows the links between each two nodes; sorted by community. (D) Heatmap that shows the intensity of each gene inside each community.

The next step was to create a network of samples. On the graph made on the similarity matrix, the best partition has a modularity of 0.47 which delineates 4 communities, that have 36, 36, 33 and respective 29 samples as can be seen in Fig 3. These four communities are different from those identified by [41]; the correspondence between our results and [41] are presented in the S6 Table.

To characterize the detected patient communities, we identified genes associated with each community. For each sample, we considered only patient-specific genes whose encoded proteins were retained in the individual network after removal of the common network and of proteins introduced during network construction. These genes originated from differentially expressed genes or genes carrying high- or moderate-impact mutations. For each community and each retained gene, we applied Fisher’s exact test to compare its frequency among samples inside the community with its frequency among samples outside the community. Genes significantly enriched in a community were retained for further pathway enrichment analysis.

The results comprise a total of 183 genes associated with the identified communities, 34 of which are shared between two clusters. Starting from these 183 genes, after excluding the pathways identified for the genes common for all networks, we obtained 15 pathways, 2 of them being shared by clusters 0 and 1 as can be seen in Table 2.

**Table 2:** Pathways identified for each cluster that were not identified for the nodes included in the common graph.

Pathway	Community
Adenylate Cyclase Activating Pathway	1
Adenylate Cyclase Inhibitory Pathway	1
Adrenaline,noradrenaline Inhibits Insulin Secretion	1
Amyloid Fiber Formation	2
Antimicrobial Peptides	2
Assembly of the ORC Complex at the Origin of Replication	0
Deposition of New CENPA-containing Nucleosomes at the	
Centromere	0 and 1
Erythrocytes Take up Carbon Dioxide and Release Oxygen	3
Erythrocytes Take up Oxygen and Release Carbon Dioxide	3
Formation of the Cornified Envelope	2
Nucleosome Assembly	0 and 1
O2 CO2 Exchange in Erythrocytes	3
Passive Transport by Aquaporins	3
Presynaptic Depolarization and Calcium Channel Opening	1
Specification of the Neural Plate Border	0

According to Reactome [42], for cluster 1, the opioid signaling might be perturbed, as well as the integration of energy metabolism, and the transmission across chemical synapses. The results showed two pathways common with cluster 0, and one of them is in fact included in the other one. They might be related to cell cycle, more precisely to nucleosome assembly by “Deposition of New CENPA-containing Nucleosomes at the Centromere.” In cluster 0, two additional potentially relevant pathways were identified. The first one, the “Assembly of the ORC Complex at the Origin of Replication” belongs to the DNA replication. The second one, belongs to the gastrulation included in the development biology.

For cluster 2, enrichment analysis identified pathways related to amyloid fibril formation, antimicrobial peptides, and keratinization. The latter is not obviously related to glioblastoma and may reflect annotation overlap or other nonspecific features of the analyzed gene set. In Cluster 3, the identified pathways included erythrocyte gas exchange and aquaporin-mediated passive transport; given their uncertain relevance to glioblastoma, these associations should also be interpreted cautiously.

Overall, our preliminary in silico analysis indicates that the four communities are associated with distinct patterns of pathway enrichment. These include processes related to GTP-dependent signaling, DNA replication and chromatin organization, protein aggregation and immune responses, and membrane transport. Because enrichment analysis identifies statistical associations rather than causal mechanisms, these community-specific patterns should be regarded as exploratory molecular hypotheses requiring further biological validation.

We investigated which of the 183 genes associated with the communities could serve as potential community-specific markers. Genes were considered as markers if their prevalence within the community exceeded 0.7, while their prevalence outside the community was below 0.3. The resulting genes are listed in the Table 3.

**Table 3:** Genes with the prevalence inside the community higher than 0.7 and prevalence outside less than 0.3.

Community	Genes
0	SOX11, MNX1, HOXD3, HASPIN, CBX2, EME1, TLX1, HAND2,
	NCAPH, MCM10, CDC25C, TFAP2A, MKI67, RAD51AP1,
	CENPH, KIF14, NUF2, PIMREG, GTSE1
1	DLGAP5, MEOX2, EXO1, ARHGAP11A, HJURP, CCNB2,
	CENPI, SKA1, KIF23, ASF1B, MELK, WDR76, KNL1, TK1
2	CHI3L1, CD163, IGFBP7, IL1R2, CSTA, ABCC3, FPR3, LOX
3	–

### Network control analyses

For each of the networks generated in the previous step, we performed a network controllability analysis to identify drug targets that might be capable of exerting control over the largest possible number of proteins on which cancer depends. The genes that encode the proteins that we want to control were identified through the integration of data from extensive RNAi loss-of-function screens and CRISPR-Cas9 knockout screens [43].

A drug is considered to exert control over an essential gene if it targets a protein capable of reaching the protein encoded by an essential gene via directed paths in the network. These paths reflect potential influence through signal transduction or regulatory cascades.

**Fig 4:**
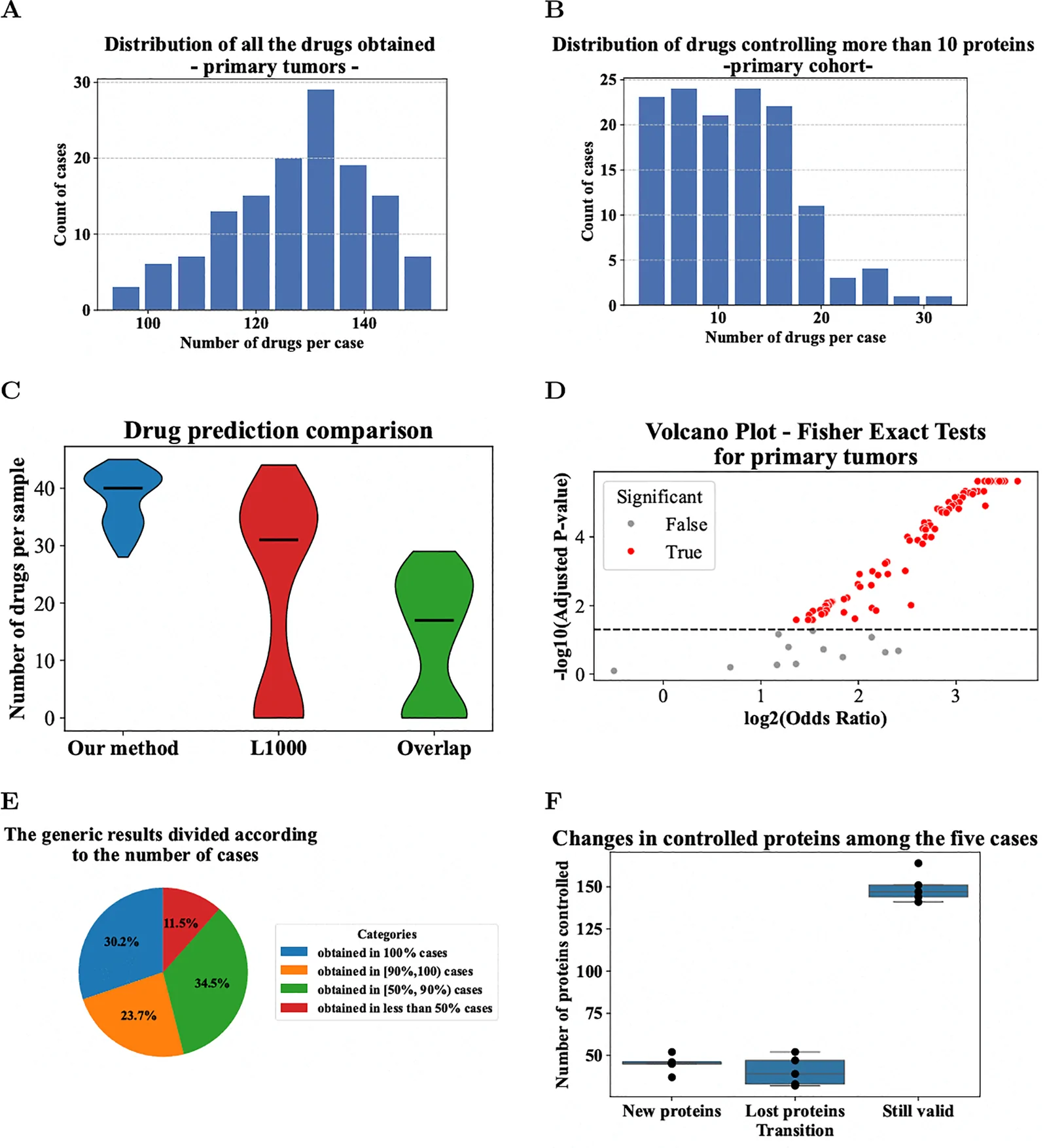
Results on the primary tumor group. (A) Number of candidate drugs identified for each primary tumor sample. (B) Number of candidate drugs capable of controlling more than 10 proteins encoded by essential genes in each primary tumor sample. (C) Distribution of the numbers of drugs identified by our method, by Enrichr, and by both methods simultaneously across primary tumor samples. (D) Volcano plot of Fisher’s exact test results comparing the drugs identified by our method and by Enrichr across primary tumor samples. (E) Comparison between drugs identified using the generic network and drugs identified across the individual primary tumor networks. (F) Differences in the numbers of essential proteins controlled by candidate drugs between primary and recurrent tumor samples for patients for whom both sample types were available.

**Fig 5:**
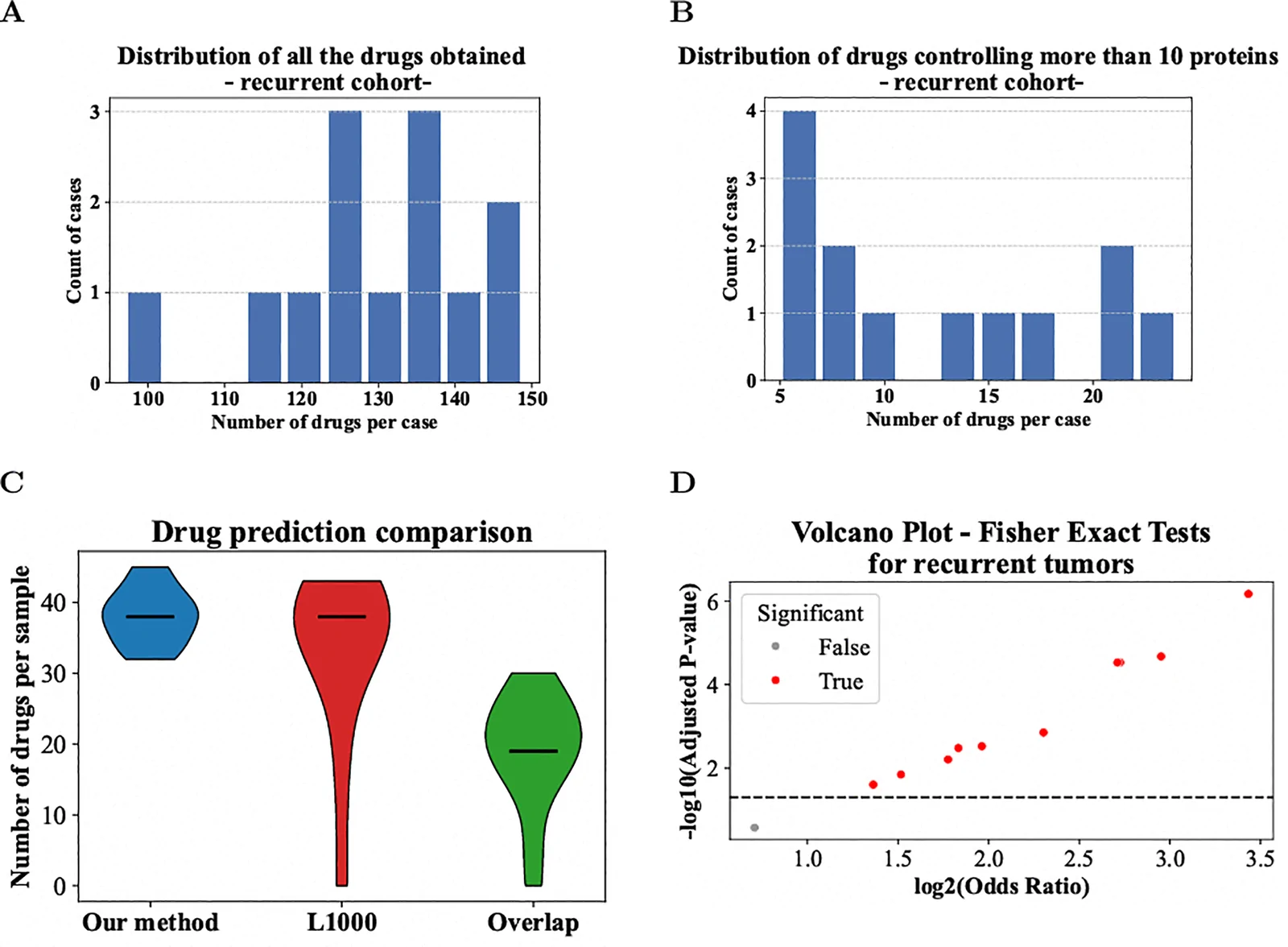
Recurrent tumor drug results. (A) The quantity of drugs identified for each recurrent tumor sample. (B) The number of drugs capable of regulating more than 10 proteins encoded by essential genes (recurrent tumors). (C) The intervals in which the drugs obtained from Enrichr, those identified by our analysis, and common drugs are located (recurrent tumors). (D) Volcano plot for Fisher Exact tests on recurrent tumor samples.

For each patient-specific network, we identified between 93 and 153 candidate drugs for primary tumors and between 97 and 149 for recurrent tumors (Figs 4A and 5A). This relatively high number of candidates can be attributed to certain proteins that are known targets of multiple approved or investigational drugs, such as EGFR (14 agents), BCL2 (4), BRAF (6), and ABL1 (7). Although many candidate drugs were identified, only a few were capable of simultaneously controlling a larger number of essential proteins.

However, if we restrict the analysis to drugs capable of targeting at least 10 essential proteins, the number of potential candidates decreases substantially. Specifically, for primary tumors, this number ranges from 2 to 33 drugs (Fig 4B), whereas for recurrent tumors it ranges from 5 to 24 (Fig 5B). In other words, although many drugs are potentially effective, only a few could be capable of simultaneously controlling a larger number of essential proteins.

For one of the analyses, taken at random, only nine drugs were found to potentially offer control over at least ten essential genes: dasatinib (affecting BCR, LCK, HSPA8, PDGFRB; controlling 13 proteins), crizotinib (MET, MST1R; 12), dimethyl fumarate (KEAP1; 11), pralsetinib (NTRK3, PDGFRB; 11), imatinib (BCR, PDGFRA, PDGFRB; 10), ponatinib (BCR, LCK, PDGFRA; 10), sunitinib and tivozanib (both targeting MET, PDGFRA, PDGFRB; 10), and zanubrutinib (LCK, TEC, BLK, ERBB4; 10).

This method, where we look for a potential control of more than 10 essential proteins, does not take all relevant factors into account. For instance, some proteins may play a more critical role in disease propagation, making it preferable to filter the list of potential drugs to include only those targeting such key proteins. Consequently, even though drugs can be ranked from a controllability perspective, we present all previously identified candidates as potential solutions rather than limiting the list.

Another important factor to consider when prioritizing candidate drugs for an individual patient is target specificity, namely whether the identified drugs act exclusively on the proteins included in our analysis or also interact with additional targets. Applying this criterion, the number of potential candidate drugs was reduced to a number per patient between 17 and 46. This requirement can be combined with the previous criterion of targeting at least 10 essential proteins. However, in this way, only a limited number of cases returned at least one option. But, when the threshold was relaxed to at least 5 essential proteins, there were between 7 and 16 options for every primary tumor case, and between 8 and 14 for every recurrent case. Based on these findings we hypothesized that drugs with high target specificity, and therefore a lower likelihood of off-target effects, might exhibit a more limited therapeutic coverage, highlighting the trade-off between target selectivity and the ability to modulate a larger set of disease-relevant proteins. However, even if a drug was obtained exclusively based on pharmacological targets, and that all such targets were identified for a given case, does not guarantee the absence of adverse reactions or side effects. To provide additional context, the S1 Table, which lists the reported side effects for a subset of the analyzed drugs, as obtained from [44]. The absence of side-effect information for the remaining drugs should not be interpreted as evidence that they don’t have adverse effects; rather, it reflects the fact that these drugs are not included in the database, and even if included, the database has not been updated in recent years. In addition, the Supplementary Material includes the S2 Table, which contains the aggregated results for all drugs obtained. For each drug identified in at least one case, there are included: (i) the subset of targets identified in at least one case in our analysis, (ii) the pharmacologically active targets identified among these according to [45], and (iii) the total number of known targets according to [45].

Since glioblastoma is located within the central nervous system, we additionally assessed the reported ability of the drugs identified in at least one analysis as possible candidate to cross the blood–brain barrier (BBB).

### Results of preliminary in silico investigations of blood-brain barrier permeability

The assessment of blood–brain barrier (BBB) permeability is a complex process that requires integration of multiple data sources. In addition to passive permeability, it is essential to consider active transport mechanisms that can significantly influence central nervous system exposure [46]. In particular, the role of P-glycoprotein (P-gp), an efflux transporter at the BBB, is critical, as it can limit brain penetration by actively exporting xenobiotics back into the systemic circulation [47]. From a pharmacokinetic perspective, optimal BBB penetration is generally associated with compounds that are not P-gp substrates, as well as those that do not substantially inhibit its function, thereby reducing the likelihood of efflux-mediated limitations and drug–drug interactions [48] However, BBB integrity may be altered in pathological conditions such as glioblastoma, where disruption of tight junctions and increased vascular permeability can modify drug distribution within the central nervous system [49].

The preliminary results of the investigation are summarized in the S3 Table. Of the 234 drugs investigated, no predictions were available for 63 compounds and there are drugs with available predictions only from admetSAR [50] or SwissAdme [51]. Based on the admetSAR analysis, 24 compounds exhibited a favorable BBB permeability profile (54 could penetrate BBB, but only 28 aren’t substrate for P-gp, and from those only 24 are not inhibitors), whereas 11 compounds were identified as favorable using SwissADME (23 can cross, but only 11 aren’t substrate). Based on these two databases, we identified only 3 common compounds that could possibly pass through the BBB: tretinoin, alitretinoin and semaxanib; for many of them the results being inconclusive.

### Benchmarking against another in silico method

We compared our model predictions using the Enrichr platform [52], specifically the L1000 database. We began by identifying drugs extracted from DrugBank (used in our analysis) included in Enrichr’s L1000 database, resulting in 109 common drugs (we considered the estradiol cypionate, estradiol valerate, estradiol benzoate as being only one compound).

By filtering the large set of drugs obtained for each sample to include only these common drugs, we reduced the number of possible candidate drugs per sample to between 28 and 45 for primary tumors, and 32–45 for recurrent tumors. Similarly, the number of drugs identified for primary tumors using Enrichr ranged from 0 to 44 per sample, while for recurrent tumors the range was 0–43. The number of drugs common to both our analysis and Enrichr varied from 0 to 29 for primary tumors, and from 0 to 30 for recurrent tumors. For the primary tumor group there were 34 out of 134 samples that had no results on Enrichr. These comparisons are presented in Figs 4C and 5C. Next, Fisher’s exact tests were performed to evaluate the association between the two methods. Resulting P-values were corrected for multiple testing using the Benjamini–Hochberg false discovery rate (FDR) procedure, and associations with adjusted P-values < 0.05 were considered statistically significant. For the cases that had results on Enrichr, only one has odds ratio below 1 and 13 have adjusted P-value more than 0.05 (Fig 4D). For the recurrent group, 1 out of 13 has no results on Enrichr, and another one has P-value above 0.05 (Fig 5D). Complete results for each case where there were solutions on Enrichr are provided in the S4 and S5 Tables.

Additionally, among the 109 common drugs, 57 drugs were never identified in our analysis for primary tumors, while 51 drugs were never identified for recurrent tumors. Similarly, 54 drugs were absent from the other method results for primary tumors, and 48 drugs were absent for recurrent tumors. Only 40 drugs for primary tumors and 35 drugs for recurrent tumors were shared between our results and the other dataset (Table 4 – Results for primary tumors).

**Table 4:** Discrepancies in cohort-level results between our drug predictions and those of the alternative in silico method.

List	Drugs
Exclusively in our set	amsacrine, cladribine, diethylstilbestrol,
	estradiol, estradiol valerate, gemcitabine,
	genistein, megestrol acetate, methotrexate,
	mitotane, paclitaxel, tacrolimus,
	tretinoin, vatalanib, vinblastine, vincristine
Exclusively by the other method	azacitidine, dactinomycin, daunorubicin,
	doramapimod, epirubicin, everolimus, idelalisib,
	lonidamine, mitoxantrone, palbociclib, PH-477736,
	quizartinib, sirolimus, vemurafenib, vindesine

Table 5 lists the drug targets through which essential proteins could be controlled by drugs identified exclusively in our analysis. Although these drugs were found in only a subset of cases, each has been cited in the literature for potential use in treating brain tumors as can be seen in Text A in S1 Appendix.

**Table 5:** Drug targets responsible for controlling essential proteins for the drugs identified only through our analysis.

Drug	Targets
amsacrine	ALB
cladribine	POLE2
diethylstilbestrol	NCOA2
estradiol	BECN1
estradiol valerate	BECN1, NCOA2
gemcitabine	CMPK1
genistein	CYP1B1, NCOA2, AKT1
megestrol acetate	PGR
methotrexate	DHFR, ATIC
mitotane	PGR
paclitaxel	BCL2
tacrolimus	FKBP1A
tretinoin	RARB, RARG, RXRA, RXRB
vatalanib	KDR
vinblastine	TUBA1A, TUBG1, JUN
vincristine	TUBA4A

Among the 422 drugs retrieved from DrugBank, including those not shared with Enrichr, only a subset appeared in our results. Some of them have targets too far away to be included in the networks. However, each network included at least 389 drug targets, corresponding to a total number of drugs equal to 406 from the initial list of 422 drugs. Ultimately, more than 150 were not obtained in any patient-specific network. On the primary tumors, only 231 were obtained in at least one primary-tumor case and 195 in at least one recurrent-tumor case. This result is expected and underscores that antineoplastic and immunomodulatory agents are not universally applicable across all cancer types.

### Primary tumor drug predictions

Based on our analysis, we identified several drugs that could be applicable across all primary cases, indicating their potential suitability for a broader patient population. These drugs were identified considering the full set of listed drugs, and not only those shared with Enrichr. The obtained set comprises 39 drugs, listed in Table A in S1 Appendix. While this number may appear large, it includes multiple compounds from the same therapeutic classes – for example, PARP inhibitors (niraparib, olaparib, rucaparib, talazoparib), HDAC inhibitors (belinostat, entinostat, panobinostat, romidepsin, vorinostat), and CDK inhibitors (alvocidib, seliciclib, trilaciclib), among others. However, only 20 of the drugs in our 39 drug set overlapped with the 109 common drugs list. Even so, when we used the *LINCS_L1000_Chem_Pert_down* dataset, no single drug from the set of 109 commonly studied compounds was applicable to all 134 cases.

To determine the clinical evaluation status of the identified compounds, we searched ClinicalTrials.gov [53] for any ongoing or completed trials involving the 39 drugs in the context of glioblastoma (Text C in S1 Appendix). Of these, 16 drugs had no associated clinical trials for glioblastoma. These include: alvocidib, antithymocyte immunoglobulin (rabbit), carfilzomib, choline, cyclosporine, decitabine, entinostat, entrectinib, estradiol acetate/benzoate/cypionate/valerate, gilteritinib, larotrectinib, midostaurin, pralsetinib, ripretinib, seliciclib, and trilaciclib. Additional details regarding these findings are provided in the Text B in S1 Appendix.

### Specificity analysis

To assess the specificity of our patient-focused analyses, we constructed a single, non-specific protein-protein interaction network by integrating all proteins encoded by genes differentially expressed at the primary tumor cohort level, as well as the protein encoded by essential genes, and drug targets. The network comprises 3363 nodes and 37928 edges, values that lie within the ranges identified for primary tumors but are positioned at the upper end of those ranges. It has a diameter of 11 and a radius of 5, like most of the individual networks. The top five nodes, ranked by importance according to the analyzed centrality measures, are listed below. As expected, for each centrality type, these nodes fall within one of the categories identified during the analysis of the individual networks presented in S3 Fig. Moreover, the corresponding individual category generally exhibits the highest percentage.

- *in-degree centrality*: UBC, TP53, SRC, EGFR, PTK2;
- *out-degree centrality*: GSK3B, CDK1, CDK2, SRC, PRKCA;
- *eigenvector centrality*: SRC, TP53, STAT3, MAPK1, UBC;
- *closeness centrality*: TP53, UBC, SRC, STAT3, CTNNB1;
- *harmonic centrality*: UBC, TP53, SRC, STAT3, CTNNB1;
- *betweenness centrality*: SRC, TP53, UBC, GSK3B, MAPK1;
- *eccentricity centrality*: STXBP2, VAMP4, KIF5C, NUP50, STX11.

Using the same controllability analysis as in the patient-specific networks, we identified drugs that might offer control over essential proteins through this generic network.

We found that only about 34.5% of the drugs identified in the generic analysis were obtained across all primary tumor cases (Fig 4E). Conversely, about 65.5% of the drugs identified in the generic network were not consistently identified across the patient-specific analyses. We also found in each patient-specific analysis between 7 and 31 candidate drugs in each patient-specific analysis that were not identified in the generic-network analysis. These findings provide preliminary evidence that patient-specific network context can alter computational drug prioritization. Furthermore, at least 5 drug targets were found exclusively in the patient-specific analyses, suggesting the importance of individual molecular context in therapeutic discovery.

We aimed to compare our findings with those from Enrichr for the generic network [52], utilizing the *LINCS_L1000_Chem_Pert_down* library on a set of 109 common drugs. Our analysis identified 41 drugs within this list, while the Enrichr results included 35 drugs, with 25 overlapping between both sets. This yields an odds ratio of 9.06 and a P-value of 7.9e-07.

### Differences between individual cases and the generic case

We sought to determine whether the differences observed between the individual cases and the generic case were driven by our analytical method or whether they persisted regardless of the method used. To address this question, and in addition to the results generated by our algorithm, we analyzed all drugs from the L1000 and NIBR datasets using Enrichr. The NIBR dataset had not been included in our previous analyses due to its limited overlap with our initial drug list. However, in the present context, where comparisons were performed within each entire dataset independently, its inclusion was appropriate. We focused specifically on up-regulated genes and identified drugs predicted to down-regulate their expression. For each dataset, we compared the results obtained for individual cases with those derived from the generic case. The analysis suggested differences across cases. Notably, this evaluation was conducted exclusively on primary tumor samples, as the recurrent group contained too few samples. To quantitatively assess the similarity between each individual case and the generic case, we calculated the Jaccard index for each comparison. Across all comparisons, we observed that the similarity was not perfect. Our algorithm provided the highest similarity and was able to provide potential predictions for all cases (S7 Fig). For our results we obtained an average Jaccard index of 0.69, while for L1000, it was 0.39, and for NIBR it was 0.21. For L1000, we had predictions only in 107 cases, while for NIBR we had only in 83 cases. There were 41 cases in L1000 and 60 cases in NIBR that had no drugs in addition to those found in the generic case. However, when there were results for a case, they were usually drugs that were not found in the generic case results, and the generic case contained drugs that were not obtained for individual cases. Because the candidate drug sets differ substantially across resources, the absolute Jaccard values are not directly comparable across datasets. Nevertheless, within each dataset, the patient-specific results differed from those obtained from the corresponding generic analysis.

### Preliminary observations of possible indicators of disease progression

Five of the patient datasets included both primary and recurrent tumor samples. For each of these patients, we constructed two separate networks: one based on the primary tumor data and the other on the recurrent tumor data. Given the small number of matched cases, this analysis was exploratory and aimed to examine whether changes between primary and recurrent tumors could be reflected in controllability-based drug prioritization. We compared the drug predictions between the two networks. Pathways unique to the primary or recurrent samples may suggest molecular changes associated with disease progression or acquired resistance, but these observations require validation in larger cohorts. The number of uniquely affected pathways for each condition is presented in Table B in S1 Appendix.

The preliminary in silico analysis showed a high degree of overlap between the controllable proteins identified in the primary and recurrent samples of all five matched cases (Fig 4F), together with case-specific differences. Given the limited number of matched samples, these observations do not allow us to determine the mechanisms underlying differences between primary and recurrent tumors. Such differences may involve transcriptional as well as other molecular or microenvironmental changes, and their interpretation will require larger cohorts and additional molecular data. These case-specific differences illustrate that primary and recurrent samples from the same patient may involve distinct molecular features.

## Discussion

We developed a personalized network-based framework integrating transcriptomic profiles, mutational data, directed protein-protein interactions, and controllability analysis to prioritize candidate therapeutics for glioblastoma. The results suggest substantial differences between patient-specific and generic networks, indicating that therapeutic opportunities identified at the individual level may not be fully captured by cohort-level analyses. These findings highlight the potential value of personalized network models for therapeutic prioritization in glioblastoma.

A central observation of this study is that patient-specific networks consistently produced different therapeutic prioritizations than generic disease networks. Although glioblastoma cases shared many molecular features, the controllability analysis identified substantial variation in the proteins that could be influenced and in the drugs predicted to affect them. This suggests that network context may play an important role in determining therapeutic opportunities and supports the use of individualized rather than disease-wide network representations.

Using the presented pipeline, we obtained patient-specific lists of computationally prioritized candidate compounds. Our exploratory analysis indicates that, for some patients, the proposed framework identifies candidate compounds that are not identified by the comparison method, illustrating its potential to reveal additional computationally prioritized options. Importantly, these compounds should be interpreted as computationally prioritized candidates rather than validated therapeutic recommendations.

The results may depend on the coverage and quality of the interaction resources used to construct the networks. Systematic database-ablation analyses constitute an interesting direction work. To assess the sensitivity of the framework to the choice of interaction resources, we compared the drug sets obtained when excluding different PPI databases (Table C in S1 Appendix). Overall, the predicted drug sets showed moderate to high agreement across several databases, particularly between KEGG, SIGNOR, and InnateDB, indicating that the proposed framework is not driven by a single interaction resource. Greater differences were observed when excluding STRING, likely reflecting its distinct coverage and interaction content. Although the choice of database influences the specific therapeutic prioritizations, these results suggest that the overall behavior of the framework remains reasonably stable across alternative sources of directed protein–protein interactions.

Sensitivity analysis of the gap parameter showed that the predicted drug sets remained largely consistent for moderate changes in the parameter value (e.g., gap = 2, gap = 3), whereas removing the gap constraint altogether (gap = 0) led to substantially different predictions SI D. This suggests that the selected parameterization represents a reasonable compromise, while also highlighting the influence of the gap constraint on the final therapeutic prioritizations.

Another sensitivity analysis was conducted to assess the effect of path length. Since our methodology requires controlling at least three nodes, a comparison with paths of length 2 was not feasible. Therefore, we applied the same criterion to paths of length 4 and 5. As the results for these longer paths were expected to be more diverse than those obtained for paths of length 3, we did not rely on the Jaccard index (Table E in S1 Appendix). Instead, we calculated the average proportion of paths that could be recovered within the longer paths. This analysis showed that, on average, 90% and 93% of the relevant paths were identified for path lengths of 4 and 5, respectively. The detailed results are provided in the Table F in S1 Appendix.

The controllability analysis may yield different solutions depending on the initial seed. However, when initialized with the same starting point, the algorithm consistently produces identical results. This was verified by performing 100 runs using the same initial point (seed 587548125), all of which generated identical solutions. Different seeds were therefore intentionally employed to explore the variability of the solutions obtained by the stochastic greedy search. To assess whether 100 repetitions provided a reasonable basis for aggregating these solutions, two preliminary studies were conducted, each comprising 100 experiments. For each experiment, we recorded the number of runs required until the first run that did not produce a previously unseen solution. The two studies yielded mean values of 85 runs (SD = 20.80 and SD = 22.13, respectively), with corresponding 95% confidence intervals for the mean of [80.87, 89.13] and [80.60, 89.40]. These consistent estimates indicate that new solutions were typically no longer observed before 100 runs under this criterion. We therefore used 100 independent runs for the analyses reported in this study. This choice is intended to reduce dependence on individual stochastic realizations rather than to imply exhaustive enumeration of all possible solutions.

The comparison performed in this study should be interpreted as computational concordance rather than biological validation. Agreement with Enrichr suggests that the proposed framework captures molecular signals also identified by an independent computational approach. At the same time, the differences between the two methods are equally informative, as they reflect distinct assumptions regarding therapeutic relevance. Additional validation using independent cohorts, pharmacogenomic datasets, perturbational experiments, or clinical response data will be required to establish predictive utility. Furthermore, pathway enrichment results may be influenced by overlapping pathway annotations, highly connected hub genes, and shared gene memberships across biological processes. Consequently, enriched pathways should be interpreted as statistical associations that suggest potential biological hypotheses rather than as direct evidence of causal disease mechanisms.

In silico community analysis identified four groups of patients characterized by distinct patterns of molecular alterations. The associated pathway enrichments suggest differences in signaling, chromatin organization, metabolism, and transport processes. However, these enrichments should be interpreted as statistical associations rather than direct evidence of distinct biological mechanisms. Similarly, the recurrent identification of proteins such as TP53, SRC, STAT3, and UBC as possible hubs in the networks is consistent with their known roles in cancer biology, although their prominence may also partly reflect their central positions within interaction networks.

The results obtained from the 134 primary tumors cases may suggest certain trends, but they cannot be generalized to the broader glioblastoma patient population without further validation in larger and more diverse cohorts. The recurrent-tumor analyses require particular caution because only 13 recurrent samples were available, including 5 matched primary–recurrent pairs. Although several potentially interesting differences between primary and recurrent tumors were identified, the limited cohort size reduces statistical power and increases uncertainty in their biological interpretation. Consequently, these findings should be regarded as hypothesis-generating observations that warrant validation in larger independent cohorts.

The proposed framework relies on several simplifying assumptions. The analysis depends on the completeness and accuracy of the underlying interaction network and is restricted to directed interactions because the controllability analysis requires directional propagation of influence. Furthermore, all high- and moderate-impact mutations are treated similarly, and proteins encoded by essential genes are used as proxies for therapeutically relevant vulnerabilities. While these assumptions enable a tractable and interpretable analysis, they may influence the resulting therapeutic prioritizations and should be considered when interpreting the results.

Additional limitations arise from the available datasets. The LINCS signatures used for comparison originate from diverse cellular contexts that do not necessarily reflect glioblastoma biology. Moreover, the transcriptomic and mutational profiles used in this study represent bulk tumor measurements and therefore do not capture the substantial spatial and clonal heterogeneity characteristic of glioblastoma. Future studies incorporating glioblastoma-specific perturbation datasets together with single-cell and spatially resolved measurements may provide a more detailed characterization of patient-specific therapeutic opportunities.

In this study, the drug search space was restricted to antineoplastic and immunomodulatory agents (ATC-L). This choice was made to focus on compounds with established relevance to cancer treatment and to maintain a manageable search space. However, potentially useful repurposing candidates from other therapeutic categories were not considered. Extending the framework to a broader spectrum of approved drugs may reveal additional therapeutic opportunities and constitutes an important direction for future work.

The primary goal of this study was not to identify universally applicable drugs, but to demonstrate that significant differences can exist between individual patient profiles and generic representations, which may substantially influence computational drug prioritization. More importantly, we aimed to provide a framework for identifying patient-specific candidate compounds for further investigation. Finally, it is worth noting that sequencing multiple samples per patient would have allowed us to capture intra-patient heterogeneity, which may also play a key role in therapeutic outcomes.

When analyzing a new primary tumor sample, up- and down-regulated genes can be identified by comparing gene expression levels to reference averages. For other cancer types, relevant databases may be used to determine genes typically dysregulated in those specific contexts. Mutated genes can also be identified and their encoded proteins incorporated into the set of disease-specific proteins.

Finally, the analysis focuses primarily on known drug targets and does not explicitly model off-target effects, pharmacokinetics, toxicity, dosage, or blood-brain barrier penetration. Consequently, the identified compounds should be viewed as computationally prioritized candidates whose clinical utility requires further evaluation.

In summary, the proposed framework demonstrates how patient-specific molecular data can be integrated with network controllability to prioritize candidate compounds in glioblastoma. While the results remain exploratory and require further validation, they illustrate the potential of personalized network models as a tool for generating clinically relevant hypotheses and guiding future experimental investigation.

## Materials and methods

### RNA-seq analyses

Our dataset was derived from the TCGA-GBM project [33], which includes 617 samples with and without gliomas. Since our focus was on samples with completed RNA sequencing, we selected 167 out of the total 617 samples. Additionally, 5 samples reported no gliomas and were thus considered as a control group. From the other group (the group containing gliomas), we excluded cases with annotations indicating pre-analysis treatment or other tumor types to avoid confounding factors in our analysis. Consequently, our final tumor dataset comprised 150 analyses, divided into two groups: 137 RNA-seq analyses performed on primary tumors and 13 RNA-seq analyses on recurrent tumors. The selection criteria are detailed in S8 Fig. Within the two tumor groups, we identified 5 patients whose samples were analyzed in both categories. For these cases, the samples were treated according to their respective groups, and differences between primary and recurrent tumors were visualized for each individual case.

### Differential gene expression analyses

We conducted a differential gene expression (DGE) analysis for the primary tumor group in comparison to the normal group, as well as for the recurrent tumor group compared to the normal group. This analysis was performed using the edgeR package [54]. We filtered for low counts and we applied the default normalization step and estimated the variance. Subsequently, we created a multidimensional scaling plot (MDS). In the MDS plot, we observed that one of the five normal samples deviated significantly from the others, as shown in S9 Fig. Consequently, we excluded this sample and repeated all the aforementioned steps. After that, differentially expressed genes in cancer versus control were identified using log fold change (lfc) set to 2 and P-value set to 0.05.

To identify genes associated with glioblastoma, differential expression analysis was first performed between tumor and control samples. The resulting set of significantly differentially expressed genes was subsequently used to construct patient-specific molecular profiles. For each significantly differentially expressed gene, the mean expression across all samples was computed. A gene was considered up-regulated in a given patient if its expression exceeded the cohort mean and down-regulated otherwise. This procedure does not constitute a patient-level differential expression analysis. Rather, cohort-level differential expression was first used to define a common set of disease-associated genes, and patient-specific expression status was subsequently assigned within this restricted set. This two-step procedure was adopted because only one tumor sample was available for each patient, precluding conventional patient-level differential expression analysis while still allowing patient-specific expression differences to be represented within a common disease-associated gene set. For each cohort-derived DEG, the expression value in an individual sample was compared with the mean expression of that gene across the corresponding tumor cohort. Given the limited number of available control samples (n = 4), the cohort mean also provided a more stable reference for this within-cohort classification than a threshold estimated exclusively from the control samples.

Since the cohort mean was used as a classification threshold, we assessed whether this strategy could introduce genes that would not have been identified by a conventional differential expression analysis against normal samples. To address this possibility, we compared the individual gene sets obtained with our approach to the results of the cohort-level differential expression analysis. No additional up-regulated genes were identified. For the down-regulated genes, 33 additional genes were detected across all individual samples; however, only six were incorporated into the interaction networks, and none were retained in the final results. These findings indicate that the individual-level classification did not substantially alter the set of biologically relevant genes included in the downstream analyses. The resulting patient-specific sets of up-regulated and down-regulated genes were combined with mutation data and used to construct the personalized protein–protein interaction networks analyzed in this study. This approach was adopted to generate patient-specific network perturbations while maintaining a common set of disease-relevant genes identified through differential expression analysis.

### Drug data

We retrieved data from the DrugBank database [45] regarding all drugs classified under the ATC code (Anatomical Therapeutic Chemical code) L, which encompasses antineoplastic and immunomodulatory agents. This approach allowed us to refine our results to those drugs applicable to various types of cancer, while simultaneously excluding those that are used in glioblastoma primarily for palliative purposes, such as antiepileptics administered during convulsions.

The initial set of extracted drugs comprised 651, but not all had known targets; specifically, only 422 did. Consequently, we refined our list to these 422 drugs, excluding potentially significant ones like temozolomide, which, despite its use in glioblastoma, lacks a known target. For each drug in the resulting list, we collected its known protein targets from DrugBank, resulting in a set of 458 unique target proteins.

### Essential genes

We define therapeutic relevance through proteins encoded by essential genes because disruption of these genes is expected to impair tumor viability. Essential genes therefore provide a biologically motivated set of candidate intervention points linking network topology to disease-relevant cellular functions.

To define the set of proteins to be controlled, we obtained glioblastoma dependencies from DepMap [43], which identified a total of 1653 genes.

After constructing the networks, we observed that the number of proteins encoded by these genes varied across different samples. Although we included all of them for each case, they were often positioned distantly from patient-specific proteins, leading to considerable variability between samples. Nevertheless, many of these proteins remained present in each network, resulting in solutions that encompass a broad range of potential drug candidates. To prioritize candidates with broader predicted influence over essential proteins, we retained only drugs with at least one target capable of controlling at least three such proteins.

### Protein-protein interactions

We gathered protein-protein interactions from various databases, including KEGG [35], OmniPath [36], Signor [37], STRING [38], and InnateDB [39]. No additional filtering based on STRING confidence scores or evidence channels was applied. However, we observed that the database applies a default confidence threshold of 150 at downloading. Directionality was the primary criterion for inclusion, as the subsequent controllability analysis requires directed interactions. Undirected interactions, including physical associations reported by several databases, were excluded because they do not specify the direction of regulatory influence. While such interactions may provide biologically relevant evidence of protein proximity or binding, they cannot be directly incorporated into the controllability framework adopted in this study. Subsequently, we mapped all resulting interactions based on Uniprot identifiers [40], yielding a list of 64,119 interactions. While this number may seem small compared to the vast human interactome referenced in other studies [55], it is crucial for our analysis to focus solely on directed interactions. Unfortunately, many experimentally identified interactions lack information regarding their directional nature.

Interactions obtained from these databases were merged into a single directed network. Duplicate directed interactions reported by multiple databases were represented only once in the final network. In cases where opposite directions of the same interaction were reported across different databases, both directed interactions were retained. The resulting network therefore represents the union of all retained directed interactions across the selected resources.

### Networks construction

We generated networks for each individual sample, as well as a network representing the overall primary tumor dataset. For each individual sample, we identified a likely specific set of proteins that included both up-regulated and down-regulated proteins, as well as proteins encoded by genes with mutations, when applicable. To this set, we added either the proteins that serve as drug targets, creating set A, or the proteins encoded by genes essential for cancer propagation, forming set B. We then considered every protein that is at a maximum distance of one, which serves as a target for arcs originating from set A or as a source for arcs leading to set B, as illustrated in S10 Fig. On the obtained networks, it is important to note that not all interactions were used in each case, as the patient-specific proteins vary slightly across cases.

Additionally, we constructed a generic network for the primary tumor, considering glioblastoma proteins as those derived from the up- and down-regulated genes identified in the differential expression analysis comparing primary tumor samples to normal samples. We excluded mutations due to their absence in all cases (for instance, high- or moderate-impact TP53 gene mutations were found in only 49 samples). Following this, we similarly created two sets by incorporating drug targets into the first set and proteins encoded by genes essential for the cancer progression into the second, after which we applied the same criteria as for the individual networks.

### Topological properties

To characterize the individual networks, as well as the generic network, we determined some topological properties. To calculate these properties, we used the NetworkX package [56], and the largest strongly connected component for each network was analyzed. The properties were: in-degree centrality, out-degree centrality, eigenvector centrality, betweenness centrality, closeness centrality, harmonic centrality, eccentricity, and its derived radius and diameter, average shortest path length, density, and average clustering. For the node-specific properties, the top five nodes from each network were compared across all networks.

### Community detection

For the primary tumor group, we aimed to determine whether the samples could be classified into distinct subgroups. We first identified the network components common to all individual patient networks. These common components represent molecular features broadly shared across the cohort and are therefore unlikely to explain differences between patients. We then removed the common network from each individual network and we excluded all the nodes that were added when constructing the networks and weren’t in the patient-specific set. Using these patient-specific proteins, we constructed a binary protein-by-sample matrix, where a value of 1 indicated the presence of a protein in a sample and 0 indicated its absence. Proteins present in very few samples or in nearly all samples were excluded, as such proteins provide little information for distinguishing between patient groups.

We then computed pairwise similarity between samples based on this filtered set of proteins. For each sample, we identified its five most similar neighbors and used these relationships to construct a sample similarity network. On this network, we applied the Louvain algorithm [57] through NetworkX to detect communities. Following community detection, we identified genes associated with each community. For each gene, we constructed a 2×2 contingency table describing its presence or absence inside and outside the community. Fisher’s exact test was used to identify genes significantly associated with each community, applying a false discovery rate (FDR) < 0.05. These community-associated genes were subsequently subjected to pathway enrichment analysis.

All significant genes, including overlapping ones, were analyzed using Enrichr to identify enriched community-specific pathways. More precisely, we searched for pathways on Reactome [42] via Enrichr [52], then we filtered the pathways to have FDR < 0.05. We made the same search for the genes belonging to the common network, using the same significance threshold of FDR < 0.05. Subsequently, we filtered the pathways identified for the genes associated with each community by excluding those that were also identified for the common gene group. We did not perform filtering across communities; consequently, some pathways were identified in more than one community.

Finally, within each community, we examined proteins with a prevalence greater than 0.7 inside the community and less than 0.3 outside, highlighting proteins that resulted in our in silico analysis to be highly characteristic of a given community.

### Networks analyses

To identify targetable proteins, we used a network controllability framework. In this setting, the protein–protein interaction network is treated as a dynamical system whose state can be steered through external inputs [58]. Here, such inputs correspond to drugs acting on specific proteins, with their effects propagating through the network via interaction pathways.

Accordingly, rather than acting directly on proteins that are critical for the cancer (referred to here as essential proteins), one can influence them indirectly by targeting upstream or intermediary proteins that regulate their behavior [59]. This perspective has two main implications. First, it removes the requirement for drugs that directly target the essential proteins, which are often unavailable [60]. Second, it enables influence over a broader subset of the network than those proteins directly bound by the drugs, due to the propagation of effects through the interaction structure [61].

Genetic disruptions at the individual level do not only affect the nodes within a network, but also the interactions between them. As a consequence, when we ran network controllability analyses, we avoided to include on the control paths the proteins encoded by down-regulated genes or by genes harboring mutations. We avoided these effects by removing, prior to the controllability analysis, interactions originating from proteins encoded by either down-regulated genes or genes harboring mutations. This choice reflects the conservative assumption that reduced expression or altered protein function may impair the ability of a protein to propagate regulatory influence through the network. We acknowledge that this simplification does not distinguish between loss-of-function and gain-of-function mutations and therefore represents an approximation of the underlying biology.

Because the greedy controllability algorithm contains a stochastic component, the analysis was repeated multiple times and the results were aggregated. This procedure reduces sensitivity to individual stochastic realizations and favors candidate drugs that are consistently identified across repeated runs. We repeated the (stochastic) network controllability analysis 100 times for each network using the NetControl4BioMed platform [62]. In this analysis, we designated drug targets as our sources, and proteins encoded by essential genes as the proteins to be controlled. We opted to apply the greedy algorithm, setting the maximum distance for control paths to three. We restricted the analysis to paths of length at most three in order to focus on relatively direct regulatory influences. This distance limit is explicitly incorporated into the algorithm, which requires each candidate drug to reach proteins encoded by essential genes at distances one, two, and three from its target. Because the interaction networks used in this study are qualitative rather than quantitative, the relative influence of paths of lengths one, two, and three cannot be estimated reliably. We therefore treated all essential proteins reachable within this distance as potentially controllable. Longer paths were excluded because they represent increasingly indirect relationships and their biological interpretation becomes less reliable in the absence of quantitative information on interaction strengths and signal propagation.

To focus on high-impact candidates, we retained only those results in which at least three essential proteins could be controlled from a drug target. Drug targets were then ranked based on the number of proteins encoded by essential genes they influenced in all 100 runs, which we used as a patient-specific network-based prioritization score.

We then identified the drugs that could act on these targets based on data from DrugBank.

### In silico comparison

We lacked experimental predictions for each individual sample and did not identify personalized results in the literature for the samples considered. To address this limitation, we leveraged data describing drug-induced effects across multiple cell lines. Several databases report genes that are up-regulated or down-regulated in response to drug treatment at specific concentrations in given cell lines. The underlying assumption is that a drug may help normalize gene expression by suppressing overexpressed genes or enhancing underexpressed ones, thereby promoting a return toward a healthy baseline state. The idea is not new; it has been explored in [63]. To compare our findings, we used Enrichr [52], specifically the *LINCS_L1000_Chem_Pert_down* library, which contains gene sets that are down-regulated by various drugs across different concentrations and cell lines. As a complementary analysis, we also investigated the underexpressed genes. However, this did not yield meaningful results, since most drugs tend to decrease gene expression and only a few induce up-regulation.

Several adjustments were necessary to align our results with those available in the Enrichr library. The first step was to harmonize the drug lists. We intersected our initial set of drugs with all drugs represented in the LINCS database, obtaining 109 drugs in common. These shared drugs were then used in subsequent analyses to identify correspondences and discrepancies on a per-sample basis.

Because the LINCS platform tests each drug at multiple concentrations – while our predictions are not concentration-specific – we considered a drug to yield a positive match if it appeared as significant at any tested concentration. On the platform, a positive result corresponds to a drug capable of reversing the overexpressed genes identified in a given sample. In practice, we submitted the list of overexpressed genes for each sample, and Enrichr computed the similarity against drug-specific down-regulated gene signatures. For each sample, the platform returned a list of drugs, each associated with a P-value and an adjusted P-value. We considered a result positive if P-value and adjusted P-value were below 0.05.

Another adjustment we made was to disregard the specific cell type on which the analysis was performed, as the available analyses were conducted exclusively in other cell lines.

We used Fisher’s exact test to assess whether the overlap between the drugs identified by our approach and those reported by the platform was greater than expected by chance, considering the 109 drugs common to both datasets. We also carried out the same comparison on the generic network.

In addition of these comparisons, in order to avoid the adjustments that need to be made when comparing ourselves to another database, we chose to compare our results obtained at the individual level with those obtained on the general network. Similarly, we proceeded with L1000, but also with NIBR. The latter being another database included on Enrichr that we used in the same way as L1000.

In addition to the in silico comparison, we conducted a review of clinical studies to determine whether the drugs identified in all the cases have been evaluated in glioblastoma or other brain cancers. We also surveyed the literature for reports on the testing of these drugs in glioblastoma or other brain malignancies, or on their activity in glioblastoma cell lines. These searches aimed to provide contextual support for our findings and to explore the potential relevance of the identified drugs across different cases, rather than to establish definitive evidence of efficacy. The results are quite diverse and they are summarized in the supplementary information.

### Preliminary in silico investigations of blood-brain barrier permeability

To evaluate the blood-brain barrier (BBB) permeability of the drugs identified in at least one patient, we first queried the admetSAR database [50] using the corresponding DrugBank entries. For each compound, we extracted the predicted BBB permeability together with the associated prediction probability. As BBB permeability alone does not necessarily reflect effective brain exposure, we also considered the predicted interaction with P-glycoprotein (P-gp), a major efflux transporter located at the BBB. Specifically, we recorded whether each compound was predicted to be a P-gp substrate, as well as its predicted classification as a P-gp inhibitor (types I and II). From the perspective of central nervous system drug delivery, the most favorable profile corresponds to a BBB-permeant compound that is neither a P-gp substrate nor a P-gp inhibitor, thereby minimizing active efflux and reducing the likelihood of transporter-mediated drug – drug interactions. Because admetSAR did not provide predictions for a substantial proportion of the investigated compounds, we retrieved their SMILES representations from PubChem [64] and analyzed them using SwissADME [51]. From SwissADME, we extracted the predictions related to BBB permeability, gastrointestinal (GI) absorption, and P-gp substrate status. SwissADME does not provide predictions regarding P-gp inhibitor classification, and therefore these data could not be included. Although GI absorption is not directly related to BBB permeability, it provides useful information regarding the suitability of oral administration. This parameter is relevant for compounds intended for oral delivery but is of limited importance for drugs administered by parenteral routes, such as intravenous injection. As an additional exploratory analysis, we estimated the likelihood of BBB penetration using physicochemical descriptors calculated with RDKit [65]. The selected descriptors included molecular weight (MW), octanol/water partition coefficient (LogP), the number of hydrogen bond acceptors (HBA), the number of hydrogen bond donors (HBD), and the topological polar surface area (TPSA), all of which are recognized determinants of BBB permeability. Descriptor calculation was not possible for approximately one quarter of the investigated compounds because SMILES representations were unavailable or could not be processed, generally due to the large size or complexity of the molecular structures. Nevertheless, this approach enabled the evaluation of three additional compounds for which predictions were unavailable from the previous tools. A heuristic scoring system was then constructed based on commonly accepted physicochemical criteria associated with BBB penetration. One point was assigned for a molecular weight below 450 Da, one point for fewer than seven hydrogen bond acceptors, and one point for fewer than three hydrogen bond donors. Lipophilicity was scored with two points for LogP values below 3.5 and one point for values between 3.5 and 5. Because topological polar surface area is considered one of the most influential determinants of BBB permeability, three points were assigned for TPSA values below 70 Å^2^, and two points for TPSA values between 70 and 90 Å^2^. In this preliminary analysis, based on descriptors, compounds with a total score greater than 6 were considered to have a favorable physicochemical profile for BBB penetration, whereas scores between 4 and 6 were interpreted as indicating moderate potential. This heuristic score was not used for the interpretation of the results but was provided in the S1 Table. However, for several compounds, contradictory predictions were obtained across different computational platforms. Therefore, we report these results as obtained, acknowledging that, in the absence of experimental validation, it remains difficult to determine which compounds are capable of crossing the BBB.

### Statistical analysis

*Differential expression analysis* was performed using the edgeR package [54] in R. Lowly expressed genes were filtered using *filterByExpr*, and library sizes were normalized using the trimmed mean of M-values (TMM) method. A generalized linear model (GLM) was fitted to compare case and baseline samples, with gene-wise dispersions estimated using the robust empirical Bayes approach (*estimateDisp, robust = TRUE*). Differential expression was assessed using the TREAT framework (*glmTreat*), specifying a minimum absolute $\log_{2}$ fold-change threshold of 2. Statistical significance was determined using *decideTests* at a significance level of 0.05. Normalized $\log_{2}$ counts per million (logCPM) values were calculated for downstream analyses, and biological coefficient of variation (BCV), multidimensional scaling (MDS), and mean-difference (MD) plots were generated for quality assessment. Following the cohort-level differential expression analysis, a custom sample-level classification procedure was applied. This procedure was intended to assign a patient-specific expression status within the set of genes already identified as disease-associated at the cohort level, rather than to perform differential expression analysis separately for each patient. For each such gene, the normalized logCPM value of an individual sample was compared with the mean value across the corresponding tumor cohort. Cohort-level up-regulated genes were classified as up-regulated in an individual sample when their expression exceeded the corresponding cohort mean, whereas cohort-level down-regulated genes were classified as down-regulated when their expression was below the corresponding cohort mean. The cohort mean was used as the primary reference because only one tumor sample was available per patient and only four baseline samples were available, making a baseline-derived sample-level threshold comparatively unstable.

To assess the robustness of this approach and ensure that sample-level expression alterations were not driven by an increased false-positive rate, results were subsequently compared with those obtained using a conventional baseline-referenced approach based exclusively on baseline samples. Therefore, following cohort-level differential expression analysis, an individual sample-level classification procedure was performed using a baseline-referenced z-score approach. For each gene identified as differentially expressed between case and baseline groups, the baseline expression distribution was characterized by calculating the mean and standard deviation of normalized logCPM values across baseline samples. The expression level of each individual case sample was then standardized relative to this baseline distribution. Genes exhibiting *z-scores* >2 were classified as sample-specific overexpression events, whereas genes with *z-scores* <−2 were classified as sample-specific underexpression events.

To identify *community-specific marker features*, each community was evaluated separately by comparing feature occurrence frequencies between samples belonging to the target community and samples from all other communities. A *one-sided Fisher’s exact test* was applied to 2 × 2 contingency tables to estimate the statistical enrichment of each feature within the respective community. Resulting P-values were adjusted for multiple comparisons using the *Benjamini–Hochberg FDR* method, with correction performed separately for each community. Features showing significant enrichment (adjusted P-value <0.05), high prevalence within the corresponding community (≥ 70%), and limited occurrence outside the community (<30%) were retained as community-specific markers.

*Cluster-level functional enrichment analysis* was conducted using Enrichr with the Reactome Pathways 2024 database. Enrichment results from individual clusters were filtered based on a Benjamini–Hochberg adjusted P-value threshold of <0.05. To distinguish cluster-specific pathway signatures from broadly shared biological processes, significantly enriched pathways overlapping with the common pathway set were excluded, and the remaining cluster-associated pathways were used for downstream analyses. Multiple testing correction was consistently performed using the Benjamini–Hochberg procedure, and statistical significance was determined based on the adjusted P-value (FDR). In cases where raw P-values were additionally considered, this did not alter the interpretation of the results, as Benjamini–Hochberg adjustment is more stringent and adjusted P-values are not expected to be lower than the corresponding raw P-values. Confidence intervals for effect size estimates were not included because the statistical analyses were designed primarily for feature selection and hypothesis testing.

Comparative distributions of the numbers of candidate drugs identified by the proposed approach, the L1000/Enrichr approach, and their intersection were summarized using violin plots. These visualizations were used for descriptive comparison and did not constitute inferential statistical analyses.

The concordance between candidate drugs identified by the proposed approach and by the Enrichr/L1000 reference method was evaluated independently for each sample using one-sided Fisher’s exact tests applied to 2 × 2 contingency tables. The contingency tables summarized the overlap, method-specific predictions, and drugs not identified by either approach within the predefined candidate drug universe. Odds ratios were calculated as measures of association, and P-values were adjusted for multiple comparisons using the Benjamini–Hochberg false discovery rate procedure. However, the primary tumor cohort-level network was evaluated as a single aggregate analysis. Statistical significance for this analysis was assessed using the nominal P-value, as no multiple comparisons were performed.

Volcano plots were generated to visualize the association between effect size and statistical significance for Fisher’s exact test results. The x-axis represents the $\log_{2}$-transformed odds ratio, indicating the strength of feature enrichment, while the y-axis represents the $-\log_{10}$-adjusted P-value, reflecting the statistical significance of the enrichment. These plots were used as descriptive visualizations to facilitate interpretation of enrichment results.

Fisher’s exact test was used to evaluate whether there was a statistically significant association between the drug sets identified by the two methods. In contrast, comparisons between the cohort-level (general) case and individual sample-level cases were performed using the Jaccard similarity index, as these analyses aimed to quantify the degree of overlap and similarity between results rather than assess statistical association. The Jaccard similarity index was also used to evaluate the consistency between networks generated under different parameter settings, including the exclusion of specific databases, variation in the number of intermediate proteins added between proteins belonging to sets A and B, and changes in the control path length. For analyses involving different path lengths, we additionally calculated the proportion of results identified at path length 3 that were retained when extending the path length to 4 or 5. This analysis was performed to assess the stability of the identified networks, considering that increasing path length is expected to expand the search space and consequently increase the number of detected results.

We further note that analyses involving pathway enrichment, community characterization, and drug prioritization were performed as exploratory, hypothesis-generating approaches. Therefore, these results are intended to provide biological interpretation and identify potential candidates for further investigation rather than serve as confirmatory evidence.

## Supporting information

S1 Fig Statistical distributions over the recurrent tumor networks. (TIF)

S2 Fig The proportion of significant nodes within a personalized network. (TIF)

S3 Fig Pie charts displaying the proportions of sequences containing the first 5 nodes obtained after applying different centrality measures on networks corresponding to primary tumors. (TIF)

S4 Fig Pie charts displaying the proportions of sequences containing the first 5 nodes obtained after applying different centrality measures on networks corresponding to recurrent tumors. (TIF)

S5 Fig Parameters of primary tumor networks. (TIF)

S6 Fig Parameters of recurrent tumor networks. (TIF)

S7 Fig Jaccard index when comparing individual case to the generic case. (TIF)

S8 Fig TCGA samples selection. (TIF)

S9 Fig One sample exclusion. (TIF)

S10 Fig The way we generated the networks. (TIF)

S1 Appendix Text A - Literature search for the drugs identified exclusively in our analysis. Text B - Literature search for the 39 drugs identified in all analyses. Text C - Clinical trials for drugs obtained across all primary tumor cases. Table A - Drugs obtained for all patients with primary tumor. Table B - Number of unique pathways identified in cases that have both primary and recurrent samples, based on proteins controlled. Table C - Mean and standard deviation of Jaccard index for the drugs obtained when excluding one protein-protein interaction database. Table D - Mean and standard deviation of Jaccard index for the drugs obtained when varying the number of proteins included between 2 proteins (=the gap) belonging to sets A or B. Table E - Mean and standard deviation of Jaccard index for the drugs obtained when varying the length of control paths. Table F - The mean and the standard deviation of the percent of the results obtained at path length equal to 3 that are included in those obtained when path length is equal to 4 or to 5.
(PDF)

S1 Table The table with side reactions extracted from SIDER. (XLSX)

S2 Table The table summarizes, for each drug, the identified drug targets, indicates which of them are pharmacologically active, and reports the total number of known targets for each drug. (XLSX)

S3 Table The table with blood-brain barrier preliminary results. (XLSX)

S4 Table Fisher values on primary tumors comparing our results with the corresponding solutions in the L1000 dataset obtained via the Enrichr platform, when available. (PDF)

S5 Table Fisher values on recurrent tumors comparing our results with the corresponding solutions in the L1000 dataset obtained via the Enrichr platform, when available. (PDF)

S6 Table The table with the correspondence between our community results and Verhaak et al. results. (XLSX)
