# Supplementary material for: In-silico personalized protein–protein interaction networks prioritize candidate compounds for glioblastoma
Source: PLoS Comput Biol. 2026 Sep 22;22(9):e1014785. doi: 10.1371/journal.pcbi.1014785 (PMC13596792; doi:10.1371/journal.pcbi.1014785)
Supplement: Supplementary file 1 [file pcbi.1014785.s011.pdf]

# Supplementary Information: In-silico personalized protein–protein interaction networks prioritize candidate compounds for glioblastoma

Nicoleta Siminea[1,2], Victor-Bogdan Popescu[3], Mihaela Păun [2,4], Ion Petre[6,2], Andrei Păun[1,5]

**1** Faculty of Mathematics and Computer Science, University of Bucharest, Bucharest, Romania
**2** Bioinformatics, National Institute of Research and Development for Biological Sciences, Bucharest, Romania
**3** Revvity Inc., Turku,Finland
**4** Faculty of Business and Administration, University of Bucharest, Bucharest, Romania
**5** Research Institute for Artificial Intelligence " Mihai Drăgănescu", Romanian Academy, Bucharest, Romania
**6** Department of Mathematics and Statistics, University of Turku, Turku, Finland

* apaun@racai.ro, ion.petre@utu.fi

## A Literature search for the drugs identified exclusively in our analysis

Amsacrine has been investigated for gliomas, both in rat glioma cell lines and in vivo models [1]. AZD-8330 has been proposed as a candidate for glioblastoma treatment [2]. Cladribine has demonstrated efficacy in glioblastoma cell lines and mouse models through a mechanism involving activation of diacylglycerol kinase B, inhibition of diacylglycerol acyltransferase 1, and increased radiosensitivity [3]. Diethylstilbestrol, historically used in prostate cancer therapy, was administered to pregnant women until its withdrawal by the FDA in 1971 due to its association with a rare vaginal cancer in female offspring [4].

Studies indicate that estradiol has a cytoprotective effect on both rat glial and neuronal cell lines [5]. At high concentrations, it can induce apoptosis and inhibit growth in these cell lines for both rats and humans [6]. Estradiol may increase survival rates in rat glial tumors [7], and, when combined with chemotherapy, it could potentially prolong survival [8]. Gemcitabine has been identified as a potential radiosensitizer in glioblastoma [9], while genistein has demonstrated a cytostatic effect in human glioblastoma cell lines [10].

Methotrexate is a drug that minimally crosses the blood-brain barrier, and, for this reason, it has been manufactured in various formulations aimed at reaching tumors. Some relevant studies include [11], where methotrexate was loaded into lipid-core nanocapsules and tested both in vitro and in vivo on rats, and [12], where methotrexate-loaded extracellular vesicles functionalized with therapeutic and targeted peptides were tested on human glioblastoma cell lines and in vivo on animals.

Paclitaxel nanoparticles have demonstrated effectiveness in glioblastoma cell lines [13]. The use of tacrolimus is suggested as a potential chemosensitizer in glioblastoma [14]. According to [15], tretinoin, a vitamin A derivative, shows no significant activity against recurrent cerebral gliomas. Vatalanib, when used in

combination with temozolomide, inhibits tumor cells more effectively than temozolomide alone [16]. Additionally, combinations such as vinblastine, mebendazol, and temozolomide may potentially address resistant cases [17]. Vincristine is included in a glioblastoma treatment regimen known as PCV (procarbazine, lomustine, and vincristine). However, lomustine is generally considered the active compound in PCV, and since vincristine does not cross the blood-brain barrier, some specialists have suggested that it should be removed from the combination [18].

# B Literature search for the 39 drugs identified in all analyses

To evaluate the relevance of the 39 drugs identified in our analysis, we reviewed the literature for evidence of their association with glioblastoma. Among these, PARP inhibitors stand out for their ability to enhance the effects of DNA alkylating agents [19]. However, the authors underlined that their effectiveness in glioblastoma is influenced by their capacity to cross the blood–brain barrier (BBB), which varies between compounds. They showed that in mouse studies, niraparib demonstrated a BBB penetration rate of 9%, compared to only 2% for olaparib [19]. This difference was confirmed in xenograft mouse models [20], where olaparib accumulated in tumors at lower concentrations than in the bloodstream and was not retained over time—unlike niraparib. Niraparib, approved for ovarian cancer, has also been shown to increase the radiosensitivity of glioblastoma cells with functional BRCA1/2 genes [21].

Olaparib, used in ovarian, breast, prostate, and pancreatic cancers, is known to have limited BBB permeability in preclinical models. Nonetheless, studies combining olaparib with temozolomide have shown its ability to penetrate both the core and periphery of recurrent glioblastoma multiforme (GBM) [22]. It also impacts cancer cell respiration and DNA repair mechanisms [23].

Rucaparib, used in ovarian and prostate cancers, has exhibited anticancer effects in GBM U251 and U87MG cells when used in combination with the PI3K inhibitor BKM120 [24]. However, its poor BBB permeability limits its therapeutic potential [25]. Similarly, talazoparib, primarily approved for breast cancer, has demonstrated efficacy in glioma stem cells both alone [26] and in combination with irradiation [27].

Several other drugs in our list also show evidence of activity against glioblastoma. Carfilzomib, a proteasome inhibitor approved for multiple myeloma, was identified by our analysis in all 134 primary cases. It significantly reduced glioblastoma cell viability [28], enhanced the efficacy of temozolomide [29], and was the most effective compound in a panel of 21 glioblastoma, 10 oligodendroglioma, and 8 astrocytoma cell lines [30].

Among the cyclin-dependent kinase (CDK) inhibitors, alvocidib was found to inhibit proliferation in U87MG, T98G, and U118MG glioblastoma cell lines [31]. Trilaciclib also shows promise, with preclinical evidence suggesting potential efficacy in aggressive cancers, including glioblastoma [32].

Belinostat, a histone deacetylase (HDAC) inhibitor, was tested in a pilot study involving 26 patients undergoing temozolomide and radiotherapy; of these, 13 received belinostat in addition to standard treatment. The results indicated improved clinical outcomes and better control of tumor recurrence in the belinostat group [33].

Cisplatin is one of the most extensively studied drugs in the context of glioblastoma, supporting its cytotoxic activity in this setting [34]. Midostaurin (CGP 41251), a multi-kinase inhibitor, has also demonstrated activity in a range of tumour cell lines, including glioblastoma [35].

Finally, two TRK inhibitors, entrectinib and larotrectinib, have shown relevance to

glioblastoma in cases involving NTRK gene fusions. Entrectinib has demonstrated efficacy in infant glioblastoma with ETV6–NTRK3 fusion [36] and in an adult case of radiation-refractory NTRK fusion glioblastoma [37]. Similarly, larotrectinib has shown activity in ETV6–NTRK3-positive infant glioblastoma [38] and a mixed response in an adult with an NTRK3 fusion tumour [39]. Durable responses have also been observed in other TRK fusion-positive glioblastoma cases [40].

Not all drugs demonstrated favorable outcomes in the context of glioblastoma. For example, choline has been shown to promote glioblastoma cell proliferation and should therefore be avoided, as evidenced in experimental studies [41].

In some cases, the findings were inconsistent. Dasatinib, for instance, was found to be ineffective in treating recurrent glioblastoma in a clinical trial [42]. However, recent in silico analyses suggest that it may have therapeutic potential, particularly in the mesenchymal subtype of the disease [43], indicating a possible context-specific effect.

Other drugs have demonstrated only limited efficacy. Tivozanib, for example, showed minimal therapeutic activity in glioblastoma according to a phase II clinical study [44], suggesting that its benefit in this setting may be restricted.

# C Clinical trials for drugs obtained across all primary tumor cases

Below is a list of the candidate compounds identified across the primary tumor cases. Only drugs currently being evaluated in clinical trials are listed. We selected trials specifically focused on glioblastoma or other types of brain cancer. When more than five clinical trials were identified, only five were included in the list. We would also like to specify that the items are listed in alphabetical order.

- **belinostat**

  NCT02137759 - “MRSI to Predict Response to RT/TMZ ± Belinostat in GBM”;

- **cisplatin**

  NCT06186440 - “Cisplatin Plus Temozolomide Compared With Temozolomide in Patients With MGMT Promotor Unmethylated Glioblastoma (Glioblastoma)”,

  NCT00003996 - “Combination Chemotherapy Plus Radiation Therapy in Treating Patients With Newly Diagnosed Glioblastoma Multiforme”,

  NCT05694416 - “Etoposide Plus Cisplatin Compared With Temozolomide in Patients With Glioblastoma”;

- **crizotinib**

  NCT02270034 - “Study to Evaluate Safety and Activity of Crizotinib With Temozolomide and Radiotherapy in Newly Diagnosed Glioblastoma”,

  NCT01441388 - “A Study Of Crizotinib Plus VEGF Inhibitor Combinations In Patients With Advanced Solid Tumors”;

- **dasatinib**

  NCT00895960 - “Dasatinib Plus Radiation Therapy/Temozolomide in Newly-Diagnosed Glioblastoma”,

  NCT00423735 - “Dasatinib in Treating Patients With Recurrent Glioblastoma Multiforme or Gliosarcoma”,

  NCT00869401 - “Dasatinib or Placebo, Radiation Therapy, and Temozolomide in Treating Patients With Newly Diagnosed Glioblastoma Multiforme”,

NCT05432518 - “GBM Personalized Trial”,

NCT00948389 - “Study of CCNU (Lomustine) Plus Dasatinib in Recurrent Glioblastoma (GBM)”;

- **dimethyl fumarate**

  NCT02337426 - “Dimethyl Fumarate, Temozolomide, and Radiation Therapyin Treating Patients With Newly Diagnosed Glioblastoma Multiforme”;

- **erdafitinib** NCT05859334 - “ Testing the Anti-cancer Drug Erdafitinib for Brain Cancers That Have Returned or Progressed Following Treatment”;

- **fingolimod** NCT02490930 - “A Safety Study of Fingolimod With Radiation and Temozolomide in Newly Diagnosed High Grade Glioma”;

- **gemcitabine**

  NCT04521686 - “Study of LY3410738 Administered to Patients With Advanced Solid Tumors With IDH1 or IDH2 Mutations”;

  NCT02711137 - “Open-Label Safety and Tolerability Study of INCB057643 in Subjects With Advanced Malignancies”;

  NCT03434262 - “SJDAWN: St. Jude Children’s Research Hospital Phase 1 Study Evaluating Molecularly-Driven Doublet Therapies for Children and Young Adults With Recurrent Brain Tumors”;

- **imatinib**

  NCT00010049 - “Imatinib Mesylate in Treating Patients With Recurrent Malignant Glioma or Meningioma”,

  NCT00039364 - “Imatinib Mesylate in Treating Patients With Gliomas”,

  NCT00021229 - “Imatinib Mesylate With or Without Radiation Therapy in Treating Young Patients With Newly Diagnosed or Recurrent Glioma”,

  NCT00171938 - “Open-label Trial of Imatinib Mesylate in Patients With Unresectable Recurrent Glioblastoma Multiforme Expressing PDGFR (Platelet Derived Growth Factor Receptors)”,

  NCT00354068 - “Imatinib Mesylate and Temozolomide in Treating Patients With Malignant Glioma”;

- **nintedanib**

  NCT01666600 - “NOA-12: BIBF1120 and R-RT in Glioblastoma”,

  NCT01251484 - “BIBF 1120 in Recurrent Glioblastoma Multiforme”,

  NCT01380782 - “BIBF 1120 for Recurrent High-Grade Gliomas”,

- **niraparib**

  NCT04715620 - “Niraparib Combined With Radiotherapy in rGBM”,

  NCT05076513 - “Trial of Niraparib in Participants With Newly-diagnosed Glioblastoma and Recurrent Glioma”,

  NCT04221503 - “Niraparib/TTFields in GBM”,

  NCT01294735 - “Study of the Safety and Efficacy of MK-4827 Given With Temozolomide in Participants With Advanced Cancer (MK-4827-014 AM1)”,

  NCT05297864 - “PARP Inhibition for Gliomas (PI-4G or *pi*4g)”;

NCT02974621 - “Cediranib Maleate and Olaparib Compared to Bevacizumab in Treating Patients With Recurrent Glioblastoma”,

NCT05463848 - “Surgical Pembro +/- Olaparib w TMZ for rGBM”,

NCT01390571 - “Olaparib and Temozolomide in Treating Patients With Relapsed Glioblastoma”,

NCT05432518 - “GBM Personalized Trial”,

NCT03212274 - “Olaparib in Treating Patients With Advanced Glioma, Cholangiocarcinoma, or Solid Tumors With IDH1 or IDH2 Mutations”,

- **panobinostat**

  NCT01115036 - “A Panobinostat Presurgery (CLBH589C)”,

  NCT05324501 - “A Study of Intra-tumoral Administered MTX110 in Patients With Recurrent Glioblastoma (MAGIC-G1)”,

  NCT00848523 - “Study of LBH589 (Panobinostat) to Treat Malignant Brain Tumors”;

- **pazopanib**

  NCT00459381 - “Pazopanib in Treating Patients With Recurrent Glioblastoma”,

  NCT02331498 - “Phase I/II Study of Pazopanib + Temozolomide in Patients With Newly Diagnosed Glioblastoma Multiforme (PAZOGLIO)”,

  NCT01931098 - “Oral Pazopanib Plus Oral Topotecan Metronomic Antiangiogenic Therapy for Recurrent Glioblastoma Multiforme (A) Without Prior Bevacizumab Exposure and (B) After Failing Prior Bevacizumab”,

  NCT00350727 - “Pazopanib In Combination With Lapatinib In Adult Patients With Relapsed Malignant Glioma (VEG102857)”,

  NCT03275558 - “Clinical Trial of the Use of the Nasal Spray of Patients With Recurrence of Glioblastoma”;

- **regorafenib**

  NCT02926222 - “Regorafenib in Relapsed Glioblastoma (REGOMA)”,

  NCT04051606 - “Regorafenib in Bevacizumab Refractory Recurrent Glioblastoma”,

  NCT04810182 - “Regorafenib in Patients With Relapsed Glioblastoma. IOV-GB-1-2020 REGOMA-OSS”,

  NCT03970447 - “A Trial to Evaluate Multiple Regimens in Newly Diagnosed and Recurrent Glioblastoma (GBM AGILE)”,

  NCT04704154 - “A Trial to Learn Whether Regorafenib in Combination With Nivolumab Can Improve Tumor Responses and How Safe it is for Participants With Solid Tumors”;

- **romidepsin**

  NCT00085540 - “FR901228 in Treating Patients With Recurrent High-Grade Gliomas”,

  NCT00053963 - “FR901228 in Treating Children With Refractory or Recurrent Solid Tumors or Leukemia”;

- **rucaparib**

  NCT02711137 - ”Open-Label Safety and Tolerability Study of INCB057643 in Subjects With Advanced Malignancies”;

- **sunitinib**

  NCT01100177 - “Study of Sunitinib Before and During Radiotherapy in Newly Diagnosed Biopsy-only Glioblastoma Patients”,

  NCT00535379 - “SUTENT (SUNITINIB, SU11248)in Patients With Recurrent or Progressive Glioblastoma Multiforme (SURGE01-07)”,

  NCT00606008 - “A Phase II Trial of Sutent (Sunitinib; SU011248) for Recurrent Anaplastic Astrocytoma and Glioblastoma”,

  NCT03025893 - “A Phase II/III Study of High-dose, Intermittent Sunitinib in Patients With Recurrent Glioblastoma Multiforme (STELLAR)”,

  NCT00864864 - “Sunitinib Tumor Levels in Patients Not on Enzyme-Inducing Anti-Epileptic Drugs Undergoing Debulking Surgery for Recurrent Glioblastoma”;

- **talazoparib**

  NCT04740190 - “Talazoparib - Carboplatin for Recurrent High-grade Glioma With DDRd (TAC-GReD)”;

- **tivozanib**

  NCT01846871 - Tivozanib for Recurrent Glioblastoma;

- **veliparib**

  NCT03581292 - “Veliparib, Radiation Therapy, and Temozolomide in Treating Patients With Newly Diagnosed Malignant Glioma Without H3 K27M or BRAFV600 Mutat”,

  NCT01026493 -“Veliparib and Temozolomide in Treating Patients With Recurrent Glioblastoma”,

  NCT02152982 -“Temozolomide With or Without Veliparib in Treating Patients With Newly Diagnosed Glioblastoma Multiforme”,

  NCT00770471 -“ABT-888, Radiation Therapy, and Temozolomide in Treating Patients With Newly Diagnosed Glioblastoma Multiforme”;

- **vinblastine**

  NCT00498979 -“Sodium Stibogluconate and IFNa-2b Followed By CDDP, VLB and DTIC Treating Pts.With Advanced Melanoma or Other Cancers”;

- **vorinostat**

  NCT01378481 - “High-Dose Vorinostat and Fractionated Stereotactic Body Radiation Therapy in Treating Patients With Recurrent Glioma”;

  NCT00238303 - “Vorinostat in Treating Patients With Progressive or Recurrent Glioblastoma Multiforme”,

  NCT00731731 - “Vorinostat, Temozolomide, and Radiation Therapy in Treating Patients With Newly Diagnosed Glioblastoma Multiforme”,

  NCT01266031 - “Phase I/II Adaptive Randomized Trial of Bevacizumab Versus Bevacizumab Plus Vorinostat in Adults With Recurrent Glioblastoma”,

  NCT01738646 - “Ph II SAHA and Bevacizumab for Recurrent Malignant Glioma Patients”,

**Table A.** Drugs obtained for all patients with primary tumor

| Drug | Our drug targets |
|---|---|
| alvocidib | CDK7, CDK9, CDK8, EGFR |
| antithymocyte Ig (rabbit) | ITGB1, ITGB3, ITGAV, CD4 |
| belinostat | HDAC1, HDAC2, HDAC3, HDAC5, HDAC6 |
| carfilzomib | PSMB2, PSMB5, PSMB8, PSMB9 |
| choline | PLD1, CHRNA7 |
| cisplatin | TF |
| crizotinib | MST1R, MET |
| cyclosporine | PPIF |
| dasatinib | BCR, BTK, KIT, LCK, HPHA5, HPHB4, HSPA8, PDGFRB |
| decitabine | HDAC1, HDAC2, HDAC3, HDAC5, HDAC6, DNMT3A, DNMT3B |
| dimethyl fumarate | KEAP1 |
| entinostat | HDAC1, HDAC2, HDAC3, HDAC5, HDAC6 |
| entrectinib | NTRK2, NTRK3, TNK2 |
| erdafitinib | FGFR1, PDGFRA, PDGFRB, KDR, KIT |
| estradiol (acetate, benzoate, cypionate, valerate) | NCOA2, BECN1 |
| fingolimod | S1PR1, S1PR3, HDAC1 |
| gemcitabine | CMPK1 |
| gilteritinib | HTR6, HTR1D, HTR1A, HTR2C, HTR1B |
| imatinib | BCR, PDGFRA, DDR2, PDGFRB, KIT |
| larotrectinib | NTRK2, NTRK3 |
| midostaurin | PDGFRA, PDGFRB, KDR, KIT |
| nintedanib | FGFR1, PDGFRA, PDGFRB, KDR, LCK |
| niraparib | PARP1 |
| olaparib | PARP1 |
| panobinostat | HDAC1, HDAC2, HDAC3, HDAC5, HDAC6 |
| pazopanib | PDGFRA, PDGFRB, KDR, KIT |
| pralsetinib | KDR, NTRK3, PDGFRB, FGFR1, JAK1 |
| regorafenib | KDR, PDGFRA, PDGFRB, FGFR1, DDR2, KIT, MAPK11 |
| ripretinib | PDGFRA, PDGFRB, KDR, KIT |
| romidepsin | HDAC1, HDAC2, HDAC3, HDAC5, HDAC6 |
| rucaparib | PARP1 |
| seliciclib | CDK7, CDK9 |
| sunitinib | PDGFRB, MET, PDGFRA, KDR, KIT |
| talazoparib | PARP1 |
| tivozanib | FGFR1, MET, PDGFRA, PDGFRB, KDR, KIT |
| trilaciclib | CDK7, CDK9 |
| veliparib | PARP1 |
| vinblastine | TUBA1A, JUN, TUBG1 |
| vorinostat | HDAC1, HDAC2, HDAC3, HDAC5, HDAC6 |

**Table B.** Number of unique pathways identified in cases that have both primary and recurrent samples, based on proteins controlled.

| Case | No. of unique pathways/primary | No. of unique pathways/recurrent |
|---|---|---|
| 1 | 80 | 8 |
| 2 | 27 | 45 |
| 3 | 50 | 56 |
| 4 | 82 | 11 |
| 5 | 13 | 35 |

**Table C.** Mean and standard deviation of Jaccard index for the drugs obtained when excluding one protein-protein interaction database.

| Excluded database versus excluded database | Mean | Standard deviation |
|---|---|---|
| InnateDB vs OmniPath | 0.502918 | 0.058096 |
| InnateDB vs SIGNOR | 0.700079 | 0.083545 |
| InnateDB vs STRING | 0.453755 | 0.056827 |
| InnateDB vs KEGG | 0.704104 | 0.085593 |
| OmniPath vs SIGNOR | 0.509585 | 0.065048 |
| OmniPath vs STRING | 0.428877 | 0.062520 |
| OmniPath vs KEGG | 0.504464 | 0.060343 |
| SIGNOR vs STRING | 0.422063 | 0.059210 |
| SIGNOR vs KEGG | 0.715591 | 0.084708 |
| STRING vs KEGG | 0.441866 | 0.053985 |

**Table D.** Mean and standard deviation of Jaccard index for the drugs obtained when varying the number of proteins included between 2 proteins (=the gap) belonging to sets A or B.

| Gap vs Gap | Mean | Standard deviation |
|---|---|---|
| Gap 1 vs Gap 2 | 0.717694 | 0.076243 |
| Gap 1 vs Gap 0 | 0.228796 | 0.079876 |
| Gap 1 vs Gap 3 | 0.718320 | 0.077132 |
| Gap 2 vs Gap 0 | 0.229161 | 0.081614 |
| Gap 2 vs Gap 3 | 0.740901 | 0.077253 |
| Gap 0 vs Gap 3 | 0.231204 | 0.084770 |

**Table E.** Mean and standard deviation of Jaccard index for the drugs obtained when varying the length of control paths.

| | | |
|---|---|---|
| Path 4 vs Path 5 | 0.718831 | 0.067734 |
| Path 3 vs Path 4 | 0.618520 | 0.071913 |
| Path 3 vs Path 5 | 0.554841 | 0.062505 |

**Table F.** The mean and the standard deviation of the percent of the results obtained at path length equal to 3 that are included in those obtained when path length is equal to 4 or to 5.

| Path | Mean | Standard deviation |
|---|---|---|
| In path 4 | 0.900324 | 0.066206 |
| In path 5 | 0.931482 | 0.057988 |

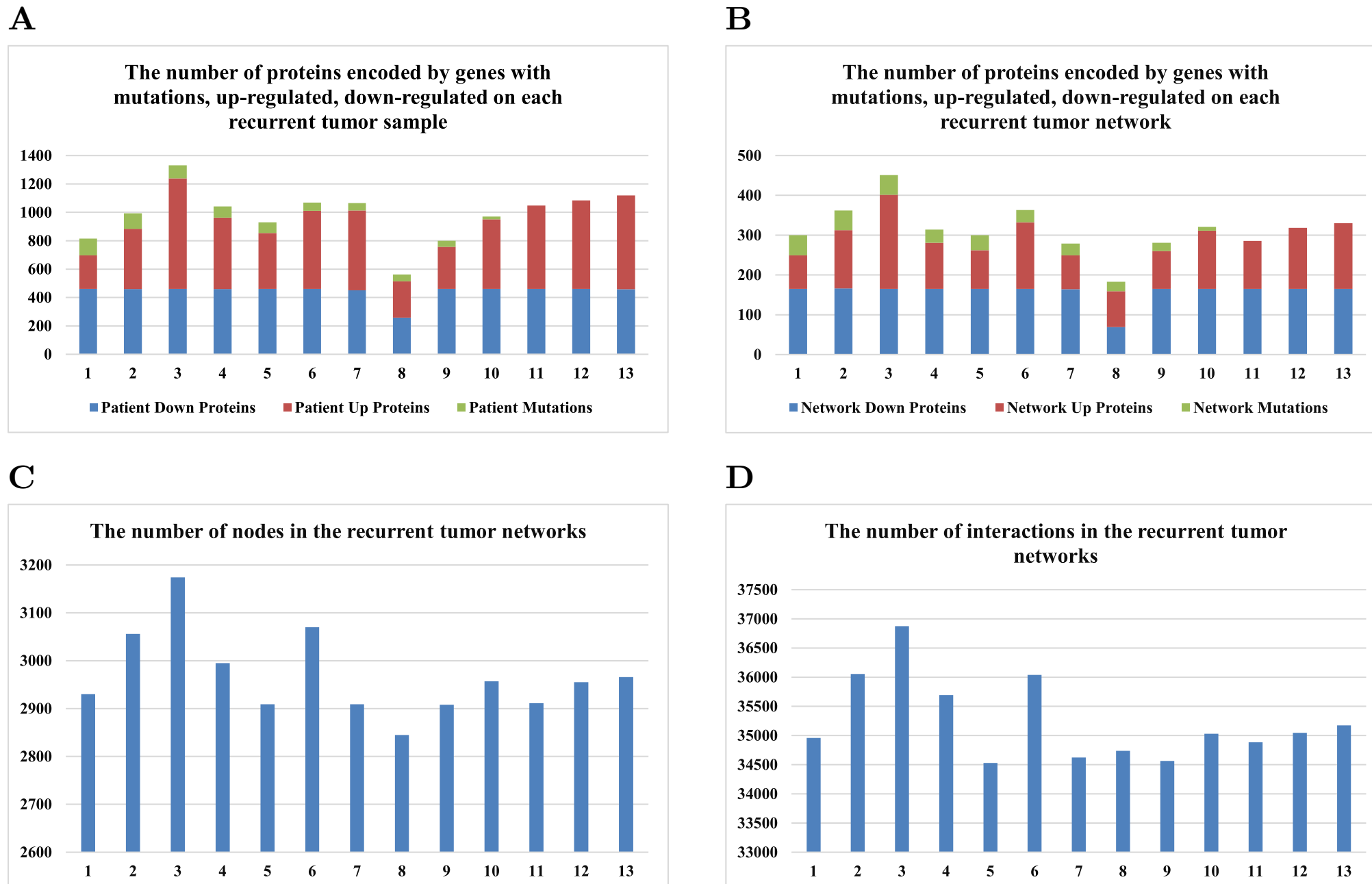


**Fig A. Statistical distributions over the recurrent tumor networks.** (A) Total sum of all specific proteins for each sample. (B) Number of specific proteins for each network generated. (C) The number of nodes within the network. (D) The number of interactions within the network.

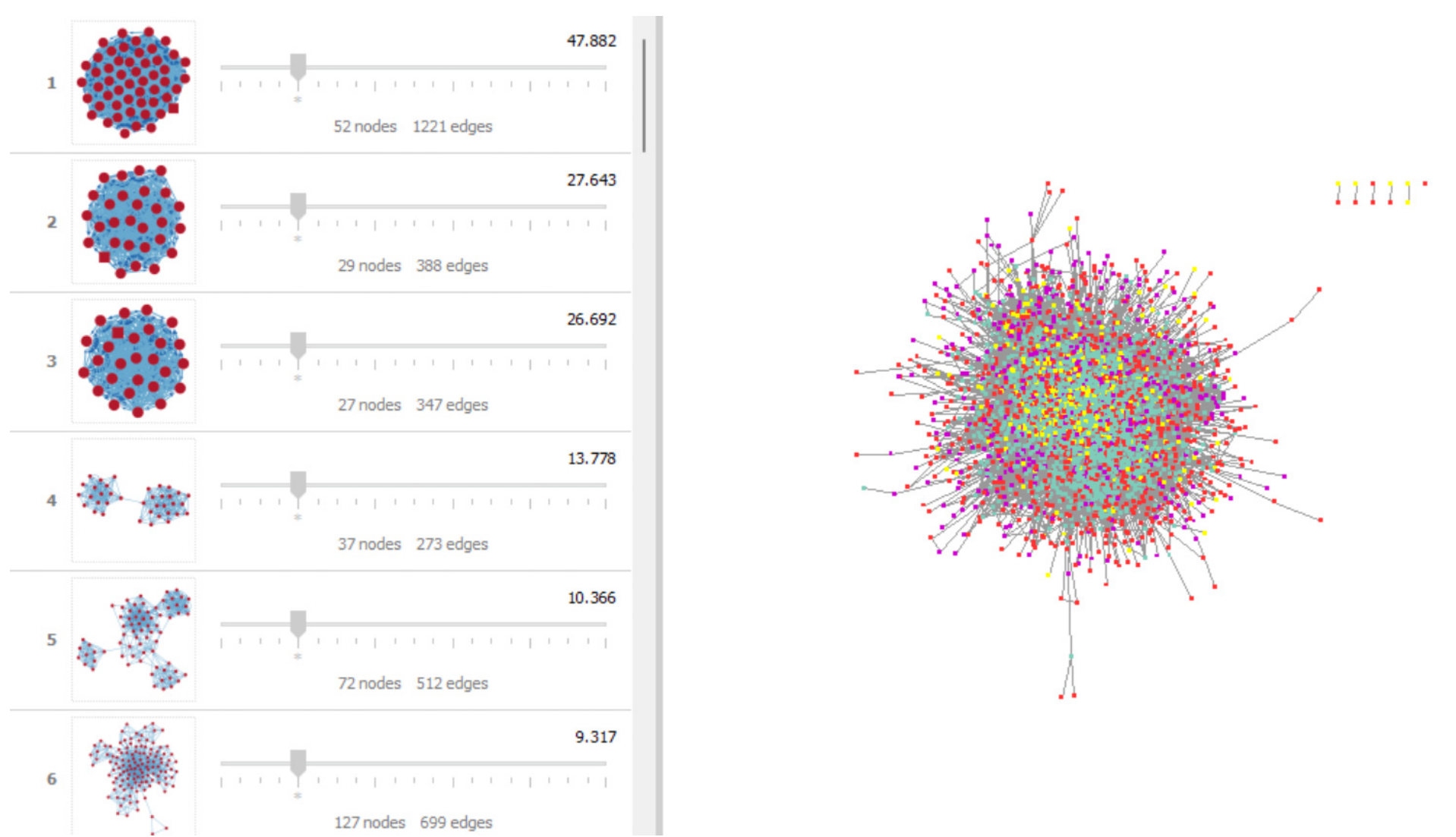


**Fig B. The proportion of significant nodes within a personalized network.** On the left: the highest-scoring five clusters identified by MCODE [45].On the right: visualization using Cytoscape [46]. Red represents essential proteins, yellow indicates drug targets that are not essential proteins, purple denotes up/down-regulated proteins or those with mutations that are neither essential proteins nor drug targets, and green-blue signifies other proteins. Starting from 825 up-regulated genes, 1002 down-regulated genes, and 53 mutations, we constructed a network comprising 3300 nodes and 37910 edges. This network includes 390 drug targets, 149 up-regulated proteins, and 392 down-regulated proteins.

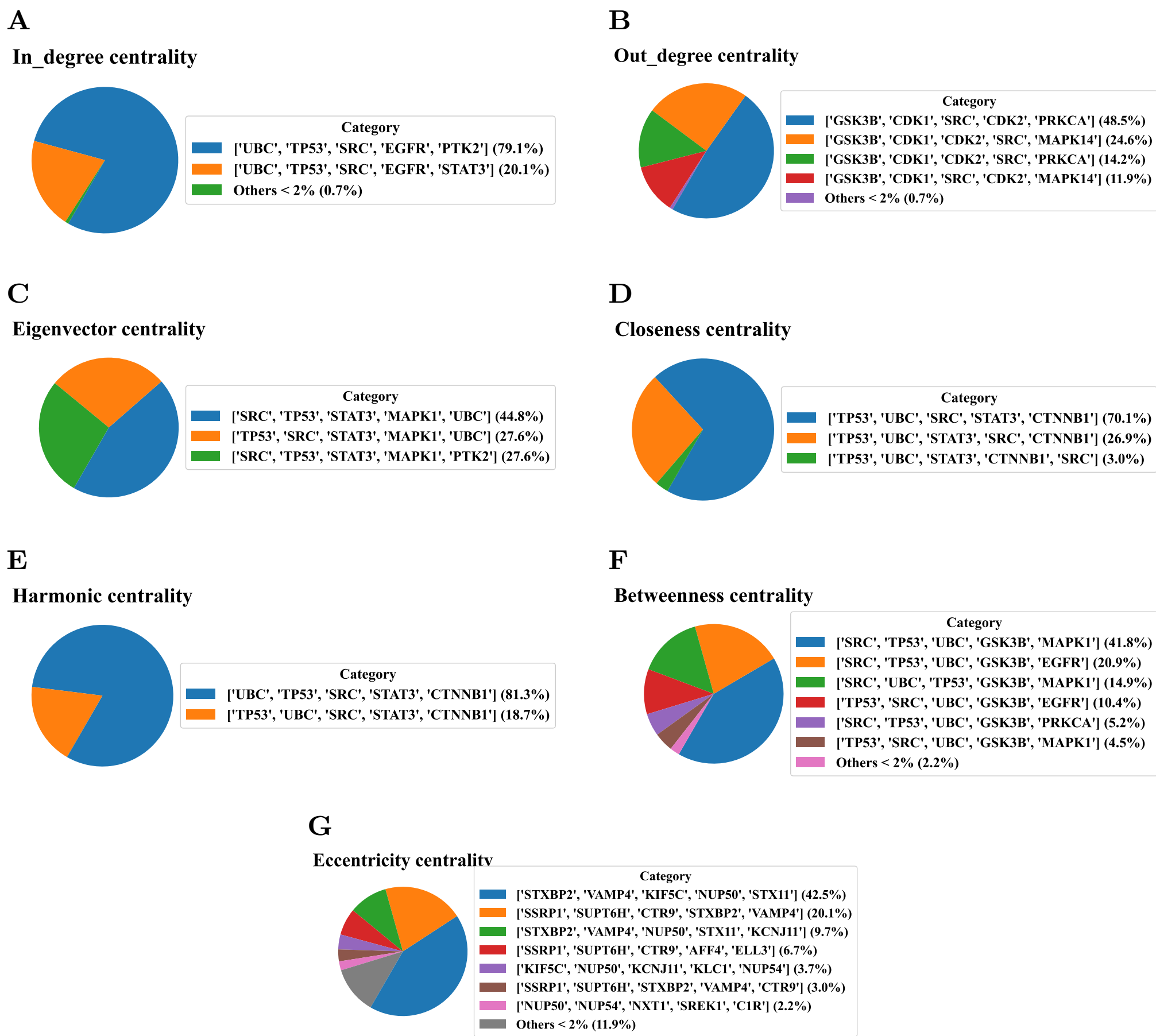


**Fig C. Pie charts displaying the proportions of sequences containing the first 5 nodes obtained after applying different centrality measures on networks corresponding to primary tumors.**
(A) In-degree centrality. (B) Out-degree centrality. (C) Eigenvector centrality. (D) Closeness centrality. (E) Harmonic centrality. (F) Betweenness centrality. (G) Eccentricity centrality.

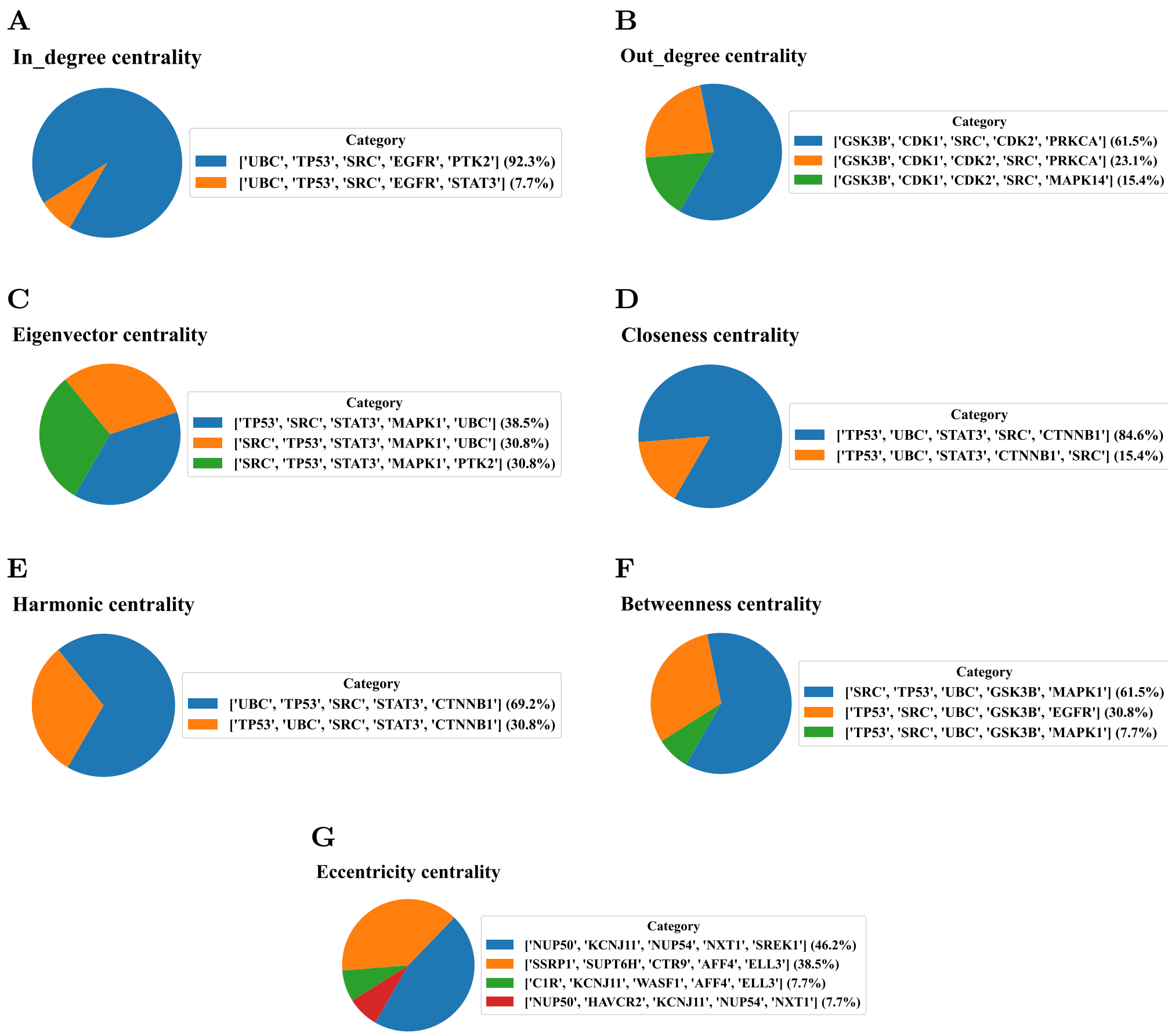


**Fig D. Pie charts displaying the proportions of sequences containing the first 5 nodes obtained after applying different centrality measures on networks corresponding to recurrent tumors.**
(A) In-degree centrality. (B) Out-degree centrality. (C) Eigenvector centrality. (D) Closeness centrality. (E) Harmonic centrality. (F) Betweenness centrality. (G) Eccentricity centrality.

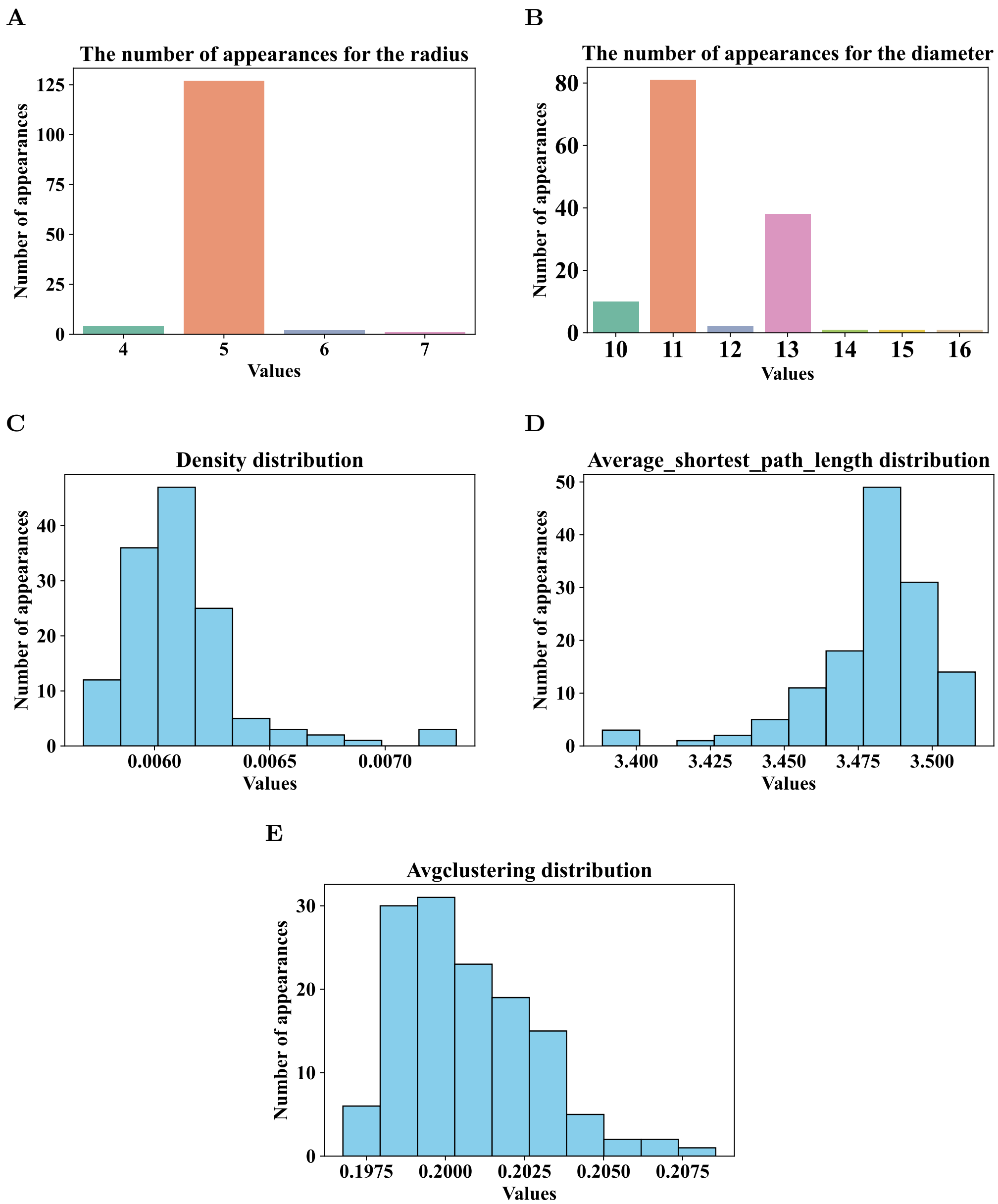


**Fig E. Parameters of primary tumor networks.**
(A) Network radius. (B) Network diameter. (C) Network density. (D) Network average shortest path. (E) Network clustering coefficient.

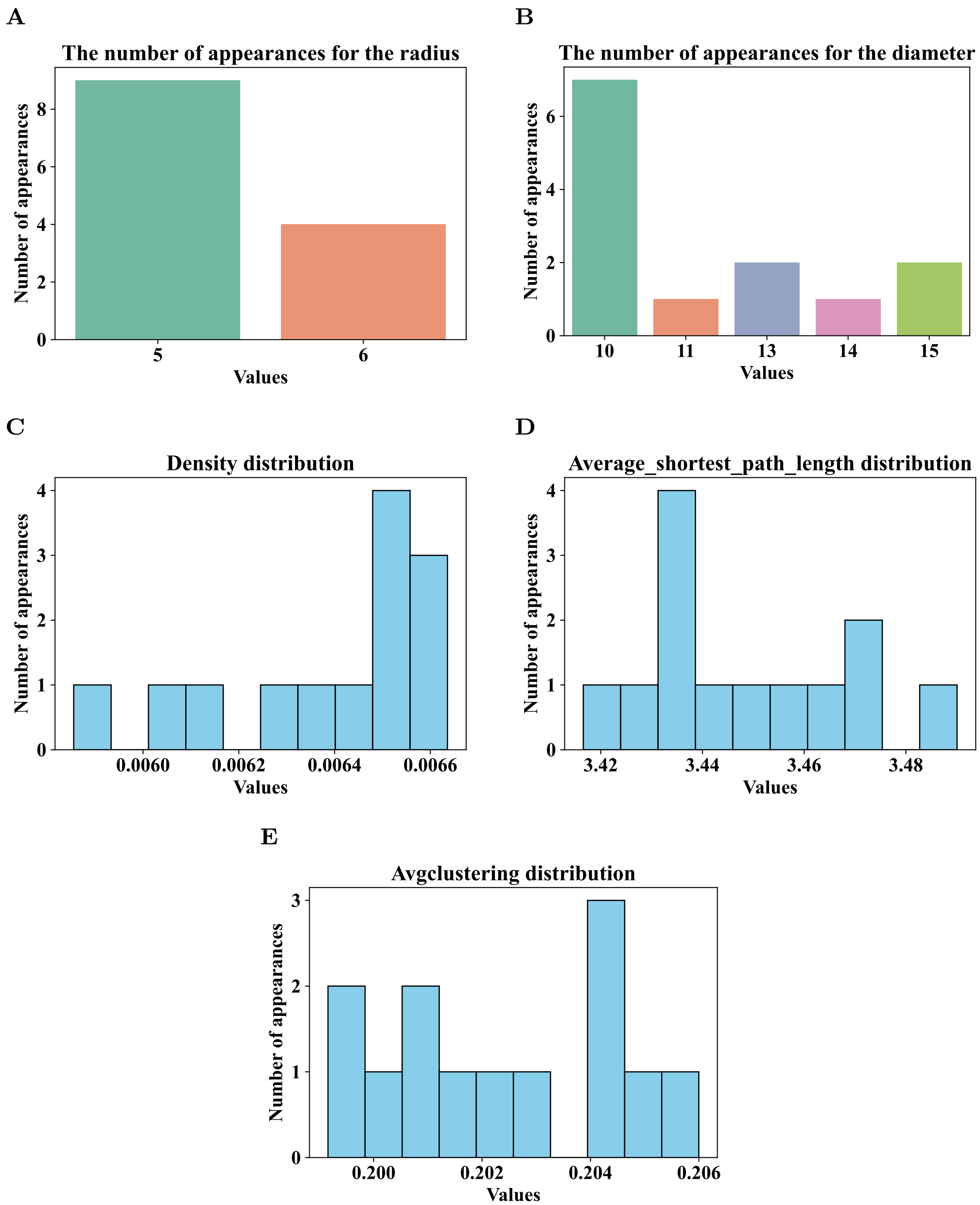


**Fig F. Parameters of recurrent tumor networks.**
(A) Network radius. (B) Network diameter. (C) Network density. (D) Network average shortest path. (E) Network clustering coefficient.

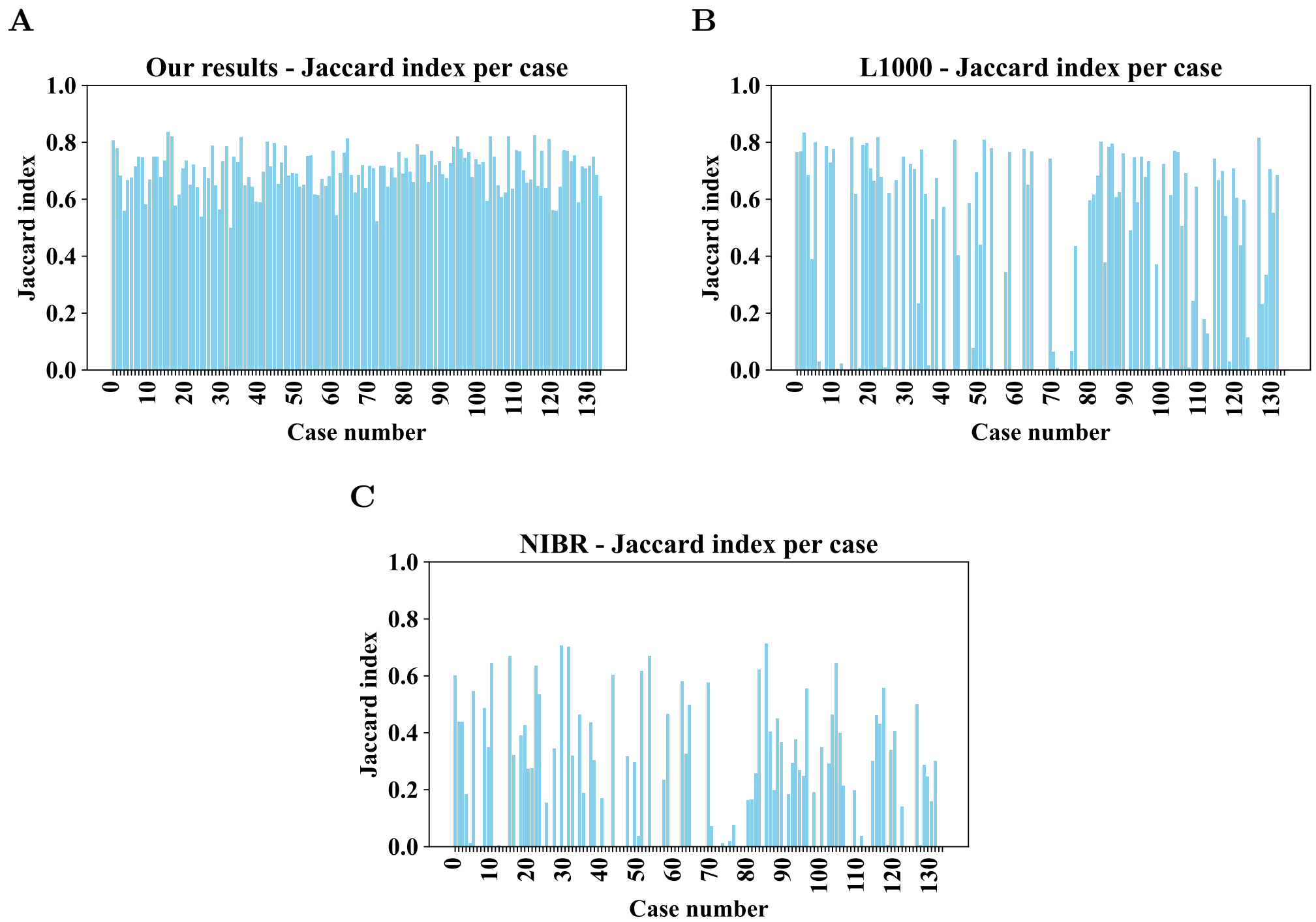


**Fig G. Jaccard index when comparing individual case to the generic case.** (A) Our results on the DrugBank subset. (B) On L1000 dataset. (C) On NIBR dataset.

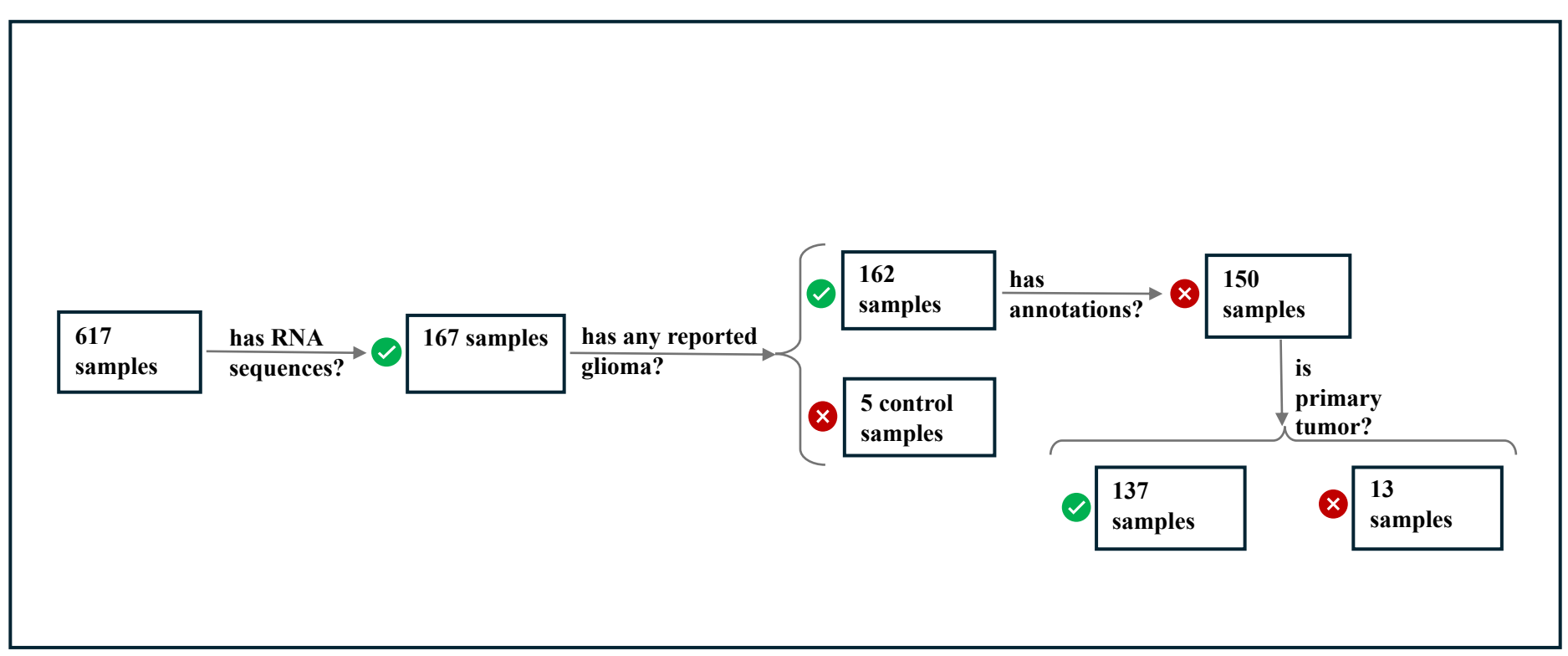


**Fig H. TCGA samples selection.**

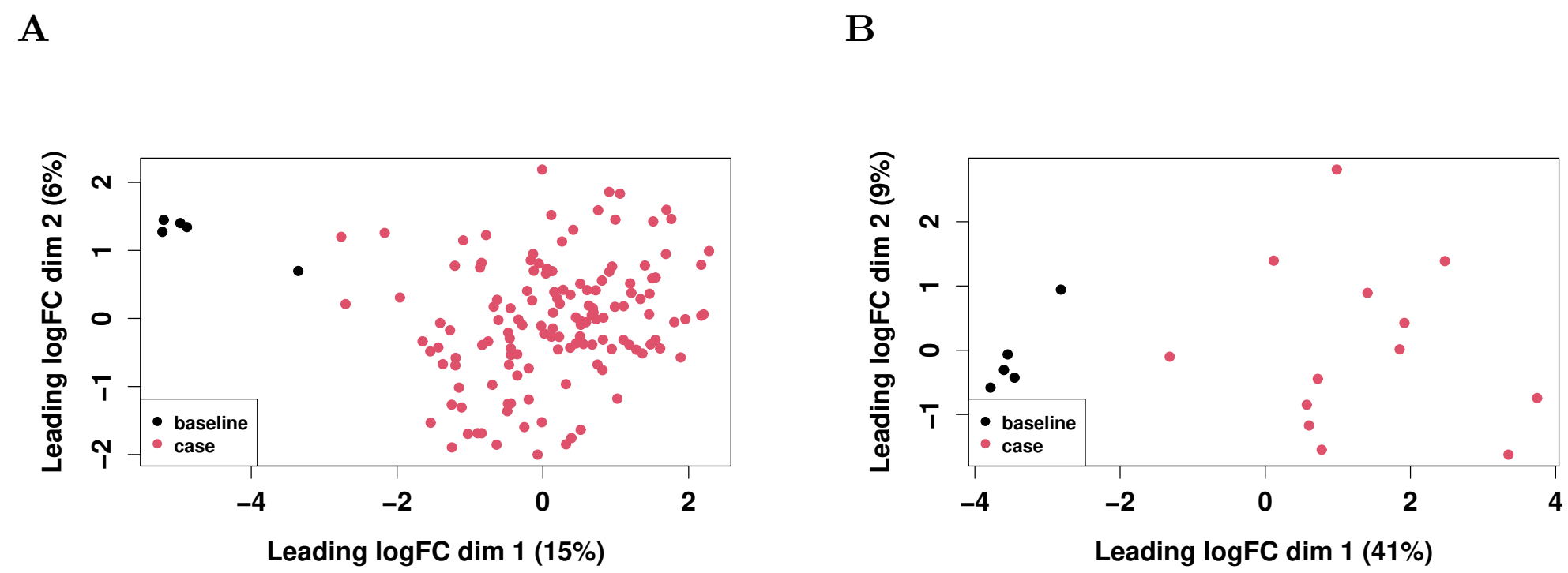


**Fig I. One sample exclusion.**
(A) MDS plot comparing the primary group and the normal group prior to sample exclusion.(B) MDS plot illustrating the recurrent group and the normal group before any sample exclusion.

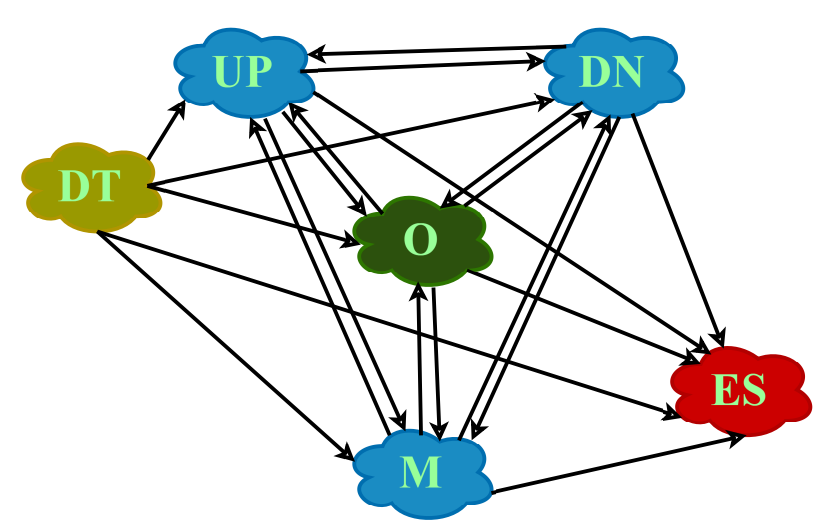


**Fig J. The way we generated the networks.**
The interaction sense is defined as follows: **DT** represents drug targets; **UP** denotes proteins encoded by up-regulated genes; **DN** indicates proteins encoded by down-regulated genes; **M** refers to proteins encoded by mutant genes; **ES** signifies proteins encoded by essential genes; **O** represents other proteins. We observe that we may have interactions such as $DT \rightarrow UP$, but also $DT \rightarrow O \rightarrow UP$; from the same UP, we can have $UP \rightarrow ES$, as well as $UP \rightarrow O \rightarrow ES$. Thus, we can reach ES through various paths, including $DT \rightarrow UP \rightarrow ES$, $DT \rightarrow O \rightarrow UP \rightarrow ES$, $DT \rightarrow UP \rightarrow O \rightarrow ES$, or even $DT \rightarrow O \rightarrow UP \rightarrow O \rightarrow ES$. It is also possible for DT to coincide with UP or even ES, leading to additional pathways.

# References


1. Wahlberg LU, Almqvist PM, Glantz MJ, Boëthius J. Polymeric controlled-release amsacrine chemotherapy in an experimental glioma model. Acta neurochirurgica. 1996;138:1323–9; discussion 1329–30. doi:https://doi.org/10.1007/BF01411063.

2. Xu H, Zhang A, Fang C, Zhu Q, Wang W, Liu Y, et al. SLC11A1 as a stratification indicator for immunotherapy or chemotherapy in patients with glioma. Frontiers in Immunology. 2022;Volume 13 - 2022. doi:https://doi.org/10.3389/fimmu.2022.980378.

3. Kang H, Lee H, Kim K, Shin E, Kim B, Kang J, et al. DGKB mediates radioresistance by regulating DGAT1-dependent lipotoxicity in glioblastoma. Cell Reports Medicine. 2023;4(1):100880. doi:https://doi.org/10.1016/j.xcrm.2022.100880.

4. Wishart DS, Feunang YD, Guo AC, Lo EJ, Marcu A, Grant JR, et al. DrugBank 5.0: a major update to the DrugBank database for 2018. Nucleic Acids Research. 2017;46(D1):D1074–D1082. doi:https://doi.org/10.1093/nar/gkx1037.

5. Bishop J, Simpkins JW. Estradiol Treatment Increases Viability of Glioma and Neuroblastoma Cells in Vitro. Molecular and Cellular Neuroscience. 1994;5(4):303–308. doi:https://doi.org/10.1006/mcne.1994.1036.

6. Altiok N, Ersoz M, Koyuturk M. Estradiol induces JNK-dependent apoptosis in glioblastoma cells. Oncology letters. 2011;2:1281–1285. doi::https://doi.org/10.3892/ol.2011.385.

7. Barone TA, Gorski JW, Greenberg SJ, Plunkett RJ. Estrogen increases survival in an orthotopic model of glioblastoma. Journal of Neuro-Oncology. 2009;95(1):37–48. doi:https://doi.org/10.1007/s11060-009-9904-6.

8. Hönikl LS, Lämmer F, Gempt J, Meyer B, Schlegel J, Delbridge C. High expression of estrogen receptor alpha and aromatase in glial tumor cells is associated with gender-independent survival benefits in glioblastoma patients. Journal of Neuro-Oncology. 2020;147(3):567–575. doi:https://doi.org/10.1007/s11060-020-03467-y.

9. Sigmond J, Honeywell RJ, Postma TJ, Dirven CMF, de Lange SM, van der Born K, et al. Gemcitabine uptake in glioblastoma multiforme: potential as a radiosensitizer. Annals of Oncology. 2009;20(1):182–187. doi:https://doi.org/10.1093/annonc/mdn543.

10. Khaw AK, Yong JWY, Kalthur G, Hande MP. Genistein induces growth arrest and suppresses telomerase activity in brain tumor cells. Genes, Chromosomes and Cancer. 2012;51(10):961–974. doi:https://doi.org/10.1002/gcc.21979.

11. Figueiró F, de Oliveira CP, Rockenbach L, Mendes FB, Bergamin LS, Jandrey EHF, et al. Pharmacological improvement and preclinical evaluation of methotrexate-loaded lipid-core nanocapsules in a glioblastoma model. Journal of biomedical nanotechnology. 2015;11:1808–18. doi:https://doi.org/10.1166/jbn.2015.2125.

12. Ye Z, Zhang T, He W, Jin H, Liu C, Yang Z, et al. Methotrexate-Loaded Extracellular Vesicles Functionalized with Therapeutic and Targeted Peptides for the Treatment of Glioblastoma Multiforme. ACS Appl Mater Interfaces. 2018;10(15):12341–12350. doi:https://doi.org/10.1021/acsami.7b18135.

13. Mu Q, Jeon M, Hsiao MH, Patton VK, Wang K, Press OW, et al. Stable and Efficient Paclitaxel Nanoparticles for Targeted Glioblastoma Therapy. Advanced Healthcare Materials. 2015;4(8):1236–1245. doi:https://doi.org/10.1002/adhm.201500034.

14. Garrido W, Muñoz M, San Martín R, Quezada C. FK506 confers chemosensitivity to anticancer drugs in glioblastoma multiforme cells by decreasing the expression of the multiple resistance-associated protein-1. Biochemical and Biophysical Research Communications. 2011;411(1):62–68. doi:https://doi.org/10.1016/j.bbrc.2011.06.087.

15. Kaba SE, Kyritsis AP, Conrad C, Gleason MJ, Newman R, Levin VA, et al. The treatment of recurrent cerebral gliomas with all-trans-retinoic acid (tretinoin). Journal of Neuro-Oncology. 1997;34(2):145–151. doi:https://doi.org/10.1023/A:1005743707803.

16. Zhang YH, Yue ZJ, Zhang H, Tang GS, Wang Y, Liu JM. Temozolomide/PLGA microparticles plus vatalanib inhibits tumor growth and angiogenesis in an orthotopic glioma model. European Journal of Pharmaceutics and Biopharmaceutics. 2010;76(3):371–375. doi:https://doi.org/10.1016/j.ejpb.2010.08.011.

17. Kipper FC, Silva AO, Marc AL, Confortin G, Junqueira AV, Neto EP, et al. Vinblastine and antihelmintic mebendazole potentiate temozolomide in resistant gliomas. Investigational New Drugs. 2018;36(2):323–331. doi:https://doi.org/10.1007/s10637-017-0503-7.

18. Weller M, Le Rhun E. How did lomustine become standard of care in recurrent glioblastoma? Cancer Treatment Reviews. 2020;87:102029. doi:https://doi.org/10.1016/j.ctrv.2020.102029.

19. Xiong Y, Guo Y, Liu Y, Wang H, Gong W, Liu Y, et al. Pamiparib is a potent and selective PARP inhibitor with unique potential for the treatment of brain tumor. Neoplasia (New York, NY). 2020;22:431–440. doi:https://doi.org/10.1016/j.neo.2020.06.009.

20. Sun K, Mikule K, Wang Z, Poon G, Vaidyanathan A, Smith G, et al. A comparative pharmacokinetic study of PARP inhibitors demonstrates favorable properties for niraparib efficacy in preclinical tumor models. Oncotarget. 2018;9:37080–37096. doi:https://doi.org/10.18632/oncotarget.26354.

21. Luo J, Xiao H, Chen Q, Li Y, Chen C, Geng M. Abstract 5720: Niraparib enhances radiosensitivity of glioblastoma cells with proficient BRCA1/2 through proliferation-related nucleolar protein DDX21. Cancer Res. 2022;82(12_Supplement):5720–5720. doi:https://doi.org/10.1158/1538-7445.AM2022-5720.

22. Hanna C, Kurian KM, Williams K, Watts C, Jackson A, Carruthers R, et al. Pharmacokinetics, safety, and tolerability of olaparib and temozolomide for recurrent glioblastoma: results of the phase I OPARATIC trial. Neuro-Oncology. 2020;22(12):1840–1850. doi:https://doi.org/10.1093/neuonc/noaa104.

23. Zampieri LX, Sboarina M, Cacace A, Grasso D, Thabault L, Hamelin L, et al. Olaparib Is a Mitochondrial Complex I Inhibitor That Kills Temozolomide-Resistant Human Glioblastoma Cells. International Journal of Molecular Sciences. 2021;22(21). doi:https://doi.org/10.3390/ijms222111938.

24. Zhang S, Peng X, Li X, Liu H, Zhao B, Elkabets M, et al. BKM120 sensitizes glioblastoma to the PARP inhibitor rucaparib by suppressing homologous recombination repair. Cell Death & Disease. 2021;12(6):546. doi:https://doi.org/10.1038/s41419-021-03805-6.

25. Majd NK, Yap TA, Koul D, Balasubramaniyan V, Li X, Khan S, et al. The promise of DNA damage response inhibitors for the treatment of glioblastoma. Neuro-Oncology Advances. 2021;3(1). doi:https://doi.org/10.1093/noajnl/vdab015.

26. Wu S, Gao F, Zheng S, Zhang C, Martinez-Ledesma E, Ezhilarasan R, et al. EGFR Amplification Induces Increased DNA Damage Response and Renders Selective Sensitivity to Talazoparib (PARP Inhibitor) in Glioblastoma. Clinical cancer research : an official journal of the American Association for Cancer Research. 2020;26:1395–1407. doi:https://doi.org/10.1158/1078-0432.CCR-19-2549.

27. Lesueur P, Chevalier F, El-Habr EA, Junier MP, Chneiweiss H, Castera L, et al. Radiosensitization Effect of Talazoparib, a Parp Inhibitor, on Glioblastoma Stem Cells Exposed to Low and High Linear Energy Transfer Radiation. Scientific Reports. 2018;8(1):3664. doi:https://doi.org/10.1038/s41598-018-22022-4.

28. Areeb Z, Stylli SS, Ware TMB, Harris NC, Shukla L, Shayan R, et al. Inhibition of glioblastoma cell proliferation, migration and invasion by the proteasome antagonist carfilzomib. Medical Oncology. 2016;33(5):53. doi:https://doi.org/10.1007/s12032-016-0767-3.

29. Unal S, Gokce T, Arslan S, Yilmaz AM, Gunduz O, Karademir B. P-355 - Combination of proteasome inhibitors with temozolomide to increase the anti-tumor effect in 3D culture model of glioblastoma. Free Radical Biology and Medicine. 2018;120:S152. doi:https://doi.org/10.1016/j.freeradbiomed.2018.04.502.

30. Wroblewski T, Tatman P, Fringuello A, Scherer S, Foreman W, Damek D, et al. DDRE-20. HIGH-THROUGHPUT SCREENING OF FDA-APPROVED COMPOUNDS IN GLIOMA AND GLIOBLASTOMA IDENTIFIES NOVEL THERAPEUTICS. Neuro-Oncology. 2021;23(Supplement_6):vi78–vi78. doi:https://doi.org/10.1093/neuonc/noab196.304.

31. Cobanoglu G, Turacli ID, Ozkan AC, Ekmekci A. Flavopiridol's antiproliferative effects in glioblastoma multiforme. Journal of Cancer Research and Therapeutics. 2016;12(2). doi:https://doi.org/10.4103/0973-1482.172132.

32. Parylo S, Vennepureddy A, Dhar V, Patibandla P, Sokoloff A. Role of cyclin-dependent kinase 4/6 inhibitors in the current and future eras of cancer treatment. Journal of Oncology Pharmacy Practice. 2019;25(1):110–129. doi:https://doi.org/10.1177/1078155218770904.

33. Xu K, Ramesh K, Huang V, Gurbani SS, Cordova JS, Schreibmann E, et al. Final Report on Clinical Outcomes and Tumor Recurrence Patterns of a Pilot

Study Assessing Efficacy of Belinostat (PXD-101) with Chemoradiation for Newly Diagnosed Glioblastoma. Tomography. 2022;8(2):688–700. doi:https://doi.org/10.3390/tomography8020057.

34. Sheleg SV, Korotkevich EA, Zhavrid EA, Muravskaya GV, Smeyanovich AF, Shanko YG, et al. Local Chemotherapy with Cisplatin-depot for Glioblastoma Multiforme. Journal of Neuro-Oncology. 2002;60(1):53–59. doi:https://doi.org/10.1023/A:1020288015457.

35. Fabbro D, Buchdunger E, Wood J, Mestan J, Hofmann F, Ferrari S, et al. Inhibitors of Protein Kinases: CGP 41251, a Protein Kinase Inhibitor with Potential as an Anticancer Agent. Pharmacology & Therapeutics. 1999;82(2):293–301. doi:https://doi.org/10.1016/S0163-7258(99)00005-4.

36. Papusha L, Zaytseva M, Druy A, Valiakhmetova A, Yasko L, Salnikova E, et al. The experience of successful treatment of ETV6-NTRK3-positive infant glioblastoma with entrectinib. Neuro-Oncology Advances. 2021;3(1). doi:https://doi.org/10.1093/noajnl/vdab022.

37. Grogan PT, Deming DA, Helgager J, Ruszkiewicz T, Baskaya MK, Howard SP, et al. Entrectinib demonstrates prolonged efficacy in an adult case of radiation-refractory NTRK fusion glioblastoma. Neuro-Oncology Advances. 2022;4(1). doi:https://doi.org/10.1093/noajnl/vdac046.

38. Alharbi M, Mobark NA, Balbaid AAO, Alanazi FA, Aljabarat WAR, Bakhsh EA, et al. Regression of ETV6-NTRK3 Infantile Glioblastoma After First-Line Treatment With Larotrectinib. JCO precision oncology. 2020;4. doi:https://doi.org/10.1200/PO.20.00017.

39. Schram AM, Taylor BS, Hechtman JF, Benayed R, Wang L, Hanusch B, et al. Abstract LB-302: Potential role of larotrectinib (LOXO-101), a selective pan-TRK inhibitor, in NTRK fusion-positive recurrent glioblastoma. Cancer Research. 2017;77(13_Supplement):LB–302–LB–302. doi:https://doi.org/10.1158/1538-7445.AM2017-LB-302.

40. Drilon AE, DuBois SG, Farago AF, Geoerger B, Grilley-Olson JE, Hong DS, et al. Activity of larotrectinib in TRK fusion cancer patients with brain metastases or primary central nervous system tumors. Journal of Clinical Oncology. 2019;37(15_suppl):2006–2006. doi:https://doi.org/10.1200/JCO.2019.37.15_suppl.2006.

41. Pucci S, Fasoli F, Moretti M, Benfante R, Di Lascio S, Viani P, et al. Choline and nicotine increase glioblastoma cell proliferation by binding and activating $\alpha$ 7- and $\alpha$ 9- containing nicotinic receptors. Pharmacological Research. 2021;163:105336. doi:https://doi.org/10.1016/j.phrs.2020.105336.

42. Lassman AB, Pugh SL, Gilbert MR, Aldape KD, Geinoz S, Beumer JH, et al. Phase 2 trial of dasatinib in target-selected patients with recurrent glioblastoma (RTOG 0627). Neuro-Oncology. 2015;17(7):992–998. doi:https://doi.org/10.1093/neuonc/nov011.

43. Alhalabi OT, Fletcher MNC, Hielscher T, Kessler T, Lokumcu T, Baumgartner U, et al. A novel patient stratification strategy to enhance the therapeutic efficacy of dasatinib in glioblastoma. Neuro-Oncology. 2021;24(1):39–51. doi:https://doi.org/10.1093/neuonc/noab158.

44. Kalpathy-Cramer J, Chandra V, Da X, Ou Y, Emblem KE, Muzikansky A, et al. Phase II study of tivozanib, an oral VEGFR inhibitor, in patients with recurrent glioblastoma. Journal of Neuro-Oncology. 2017;131(3):603–610. doi:https://doi.org/10.1007/s11060-016-2332-5.

45. Bader GD, Hogue CWV. An automated method for finding molecular complexes in large protein interaction networks. BMC Bioinformatics. 2003;4(1):2. doi:https://doi.org/10.1186/1471-2105-4-2.

46. Smoot ME, Ono K, Ruscheinski J, Wang PL, Ideker T. Cytoscape 2.8: new features for data integration and network visualization. Bioinformatics. 2010;27(3):431–432. doi:https://doi.org/10.1093/bioinformatics/btq675.
