# Supplementary material for: In-silico personalized protein–protein interaction networks prioritize candidate compounds for glioblastoma
Source: PLoS Comput Biol. 2026 Sep 22;22(9):e1014785. doi: 10.1371/journal.pcbi.1014785 (PMC13596792; doi:10.1371/journal.pcbi.1014785)
Supplement: Supplementary file 2 [file pcbi.1014785.s015.pdf]

Fisher test results for primary tumors

Sample	Odds ratio	p value	Adjusted P-value	Significant
01521666-f595-4074-aea1-f7ab78db062b	12.38095238	3.37E-08	2.36087E-06	TRUE
19f1375f-51f2-4f4c-88fe-6fc84c37e5b6	11.31617647	8.94E-08	2.36087E-06	TRUE
b25c8e4d-f6ca-4079-9120-b96147b9091e	10.6875	1.24E-07	2.36087E-06	TRUE
a5525401-79be-4c7a-9107-71c022778d65	9.792207792	2.73E-07	2.36087E-06	TRUE
a504b1cd-7d40-40e8-8d58-267c680768e5	9.321428571	2.46E-07	2.36087E-06	TRUE
820e3299-bd67-4ba8-a637-32d31205e120	10.71428571	1.55E-07	2.36087E-06	TRUE
6836d774-e17b-4198-b22a-47d05819b05d	10.24305556	2.83E-07	2.36087E-06	TRUE
53d78258-1650-4793-a01d-d2c065215fb9	10.84444444	2.03E-07	2.36087E-06	TRUE
b0717300-0735-4451-bba1-7ce0fc0d4ac6	11.08823529	1.34E-07	2.36087E-06	TRUE
d984a0e2-d502-4003-bdbf-a0364d4e5a78	10.24305556	2.83E-07	2.36087E-06	TRUE
c1fdf09e-3960-4aaa-8c49-fe64c6c5bcea	10.05333333	2.34E-07	2.36087E-06	TRUE
bd4b6975-90d9-43f8-aadc-344d04644822	10.88888889	8.5E-08	2.36087E-06	TRUE
419b4c0a-c23f-4214-8de0-722d2ae0846b	8.538888889	5.97E-07	4.59208E-06	TRUE
adce8dd4-e2df-43ed-9821-3781ddb7a82f	9.350649351	6.5E-07	4.64583E-06	TRUE
4befb3ab-b606-4035-a0b0-639032962767	9.0625	7.9E-07	4.64754E-06	TRUE
37889f67-62a8-483b-97af-d8391806ede9	9.743055556	7.17E-07	4.64754E-06	TRUE
c42fa4ad-4f31-4dfd-b499-599896a57517	9.0625	7.9E-07	4.64754E-06	TRUE
020fcc42-97ff-4959-b52f-f7f119c3f643	8.787878788	9.44E-07	5.18739E-06	TRUE
2b421eac-4980-4b9c-bddd-ad0818ec15e6	8.4375	9.86E-07	5.18739E-06	TRUE
bcdc68db-c874-4097-9c46-b06e331caaf5	8.993589744	1.12E-06	5.60329E-06	TRUE
ed9fe516-13b7-45d1-8d17-40ca9a96bbd0	8.382352941	1.68E-06	7.00004E-06	TRUE
d90b02f3-4c68-40ec-9704-1d1d6706e402	8.088888889	1.61E-06	7.00004E-06	TRUE
c6045584-592e-412f-974a-c8ae2d4bb817	8.382352941	1.68E-06	7.00004E-06	TRUE
fcc54ed3-5ba9-461f-a3a5-72b8eb89e4da	7.935828877	1.57E-06	7.00004E-06	TRUE
dfa2ddfb-c161-4c8c-a538-be6e00a8466e	7.59375	2.52E-06	9.68879E-06	TRUE
39701843-3dae-4114-b04c-b3387e402e4f	8.188235294	2.52E-06	9.68879E-06	TRUE
7cc30f38-a19e-4552-a5ca-64a11575abed	7.909090909	3.07E-06	1.13746E-05	TRUE
afe67ba6-d59f-4450-8fbd-624da67d47e5	7.777777778	3.45E-06	1.23092E-05	TRUE
c8460566-8199-4a4d-93fd-67692611992d	9.841269841	3.58E-06	1.23399E-05	TRUE
bcbb79d8-1d4a-4fbb-b16c-4df86839773e	8.148148148	4.67E-06	1.50627E-05	TRUE
30e10839-2ba1-4fd1-82ed-ad8fe4e72794	7	4.53E-06	1.50627E-05	TRUE
1ed5fd9b-a682-4e81-bda1-7ed085f4355d	7.6	5.06E-06	1.53386E-05	TRUE
a75c6098-ff48-43c2-b56d-6fa448ddebfb	7.6	5.06E-06	1.53386E-05	TRUE
982e2940-10b0-4383-bde8-f7ccb9920fc4	7.179487179	5.49E-06	1.61586E-05	TRUE
02654be6-2049-4000-a0ac-c26f7ba6f0c9	7.315508021	6.29E-06	1.79756E-05	TRUE
284d5f4a-5b44-42f7-90e6-14a5250183aa	7.236842105	6.84E-06	1.89968E-05	TRUE
2fb19a94-bd55-4041-994a-082cd30746f3	7.466666667	7.28E-06	1.96699E-05	TRUE
b888bce3-6b9e-494b-b1e8-13714c8b5267	6.588235294	1.48E-05	3.8262E-05	TRUE
cbdf8c09-e3f4-4b3e-bdaa-f4a3a4e40068	6.37962963	1.49E-05	3.8262E-05	TRUE
bd7b2c7c-3a38-4a2b-b6eb-5f67572db883	6.656410256	1.82E-05	4.55505E-05	TRUE

3fca2c7c-2541-418d-8f45-2a48814ca574	6.426470588	2.16E-05	5.26536E-05	TRUE
b38a841a-f57e-4170-8cbc-17c54aacd580	6.315789474	2.38E-05	5.66672E-05	TRUE
f6a99d72-7ef2-4c9c-880c-a26bd5fef9c7	6.883333333	2.51E-05	5.84207E-05	TRUE
b004e38d-f48c-49f9-a027-4a702fbaf107	6.44	2.68E-05	6.08553E-05	TRUE
f2d5627a-f891-453b-8bdb-38d732c59342	6.444444444	4.4E-05	9.78519E-05	TRUE
7f7121ff-1399-40da-a792-b7c31bc76570	5.662393162	4.57E-05	9.93451E-05	TRUE
dc3875c2-1a88-491c-bf98-fa344dd3b07e	6.704545455	4.74E-05	0.000100929	TRUE
04be0c6b-c75f-40cf-82c0-c14af4f9e456	6.09	5.93E-05	0.000123556	TRUE
8a395eef-a9cc-4f21-b8d2-aefa292077cf	5.75	6.22E-05	0.000126901	TRUE
4d4a1165-e304-4aed-9efa-7f6f5bacdb5e	6.304347826	7.88E-05	0.000157697	TRUE
44ed61ae-e74e-40d7-98ce-7803e040567f	4.9	0.000272	0.000534047	TRUE
600932fb-d31e-4b69-afe7-e3803513d71f	4.833333333	0.000309	0.000594616	TRUE
3f171964-37b4-43a2-b2f9-1b51dd924a7e	5.577142857	0.000516	0.000972648	TRUE
4612a611-2ff9-4f51-b4b2-af142254dbe8	4.416666667	0.00054	0.000999758	TRUE
cbc1bf1c-8ddd-424a-b95a-5ca231d94e63	4.927536232	0.000676	0.001207607	TRUE
28f0c53a-abdd-4789-adcf-f59132ae3a92	4.029605263	0.000676	0.001207607	TRUE
8af78255-e426-4a5d-a997-84946f5e459c	4.597402597	0.000734	0.001287245	TRUE
2f7160fe-5f34-49bc-95fd-029ce83d8c42	3.981617647	0.001382	0.002383044	TRUE
ab5f9e4e-ff6a-4353-83f4-5ffddca0050a	4.382222222	0.001503	0.002547985	TRUE
6508275a-e712-424e-bdaf-b9e1b07b0f95	4.047619048	0.001708	0.002846836	TRUE
746ed118-eb9f-43a5-bcda-872999ae7128	3.692307692	0.003624	0.005941445	TRUE
06227615-f651-462b-948f-c305ba369efa	3.609022556	0.004019	0.006482274	TRUE
a5461e7c-620e-49c0-b4bf-f9774b9393af	3.315789474	0.005022	0.007846749	TRUE
1a860864-2ba1-46ad-ad36-018a7b0ec08e	3.315789474	0.005022	0.007846749	TRUE
2afdb646-75ca-4bc9-9c12-30a27f994ecd	3.294117647	0.00518	0.007968649	TRUE
f47c3aa6-0f0d-4e0d-b6cf-bf565d511d6d	3.20625	0.005577	0.00832443	TRUE
02d43414-c8c9-42d3-8009-f9fb164dc8e0	3.20625	0.005577	0.00832443	TRUE
21800024-cf76-4185-b57b-526539ccdba2	5.80952381	0.006642	0.009767188	TRUE
e9a0fc7b-94ea-477d-918a-1ef80fefefcd	3.24	0.006744	0.009773233	TRUE
2c15cc20-2347-4bfc-826d-a062f6d74b46	3.1875	0.007081	0.010116007	TRUE
378f6079-6c4e-415b-8b5e-6c3c72e513b3	3.166666667	0.007295	0.010132427	TRUE
2a254376-7498-4c41-8c20-8b4bdd8369da	3.166666667	0.007295	0.010132427	TRUE
ea50d2da-4f84-46dc-8036-c34392793aed	4.4	0.008576	0.011748114	TRUE
64718111-dba7-4f49-9f3a-7f2bccc12cda	3.093333333	0.00953	0.012878667	TRUE
da09dd77-0b91-4795-bd80-f90a9784b65c	3.052631579	0.010097	0.013462001	TRUE
44067d6e-af12-4abf-8795-cb6749b1cc7a	3.191176471	0.010272	0.013515361	TRUE
0f12d09f-f512-4a6a-95e2-c8942488f2ff	4.533333333	0.0108	0.014025845	TRUE
3d4081b3-24c9-4dbd-a5fe-0fb29ce43737	2.892857143	0.011226	0.014392443	TRUE
ce3cc39f-d031-4751-a9fc-d9c7b2fb168f	3.60591133	0.01253	0.015860988	TRUE
742b41f9-faca-4bad-a2a5-5d40b8bf06ce	3.15	0.013244	0.016555319	TRUE
bdba7172-5280-41f6-8082-dbf8983c4089	3.078947368	0.01448	0.017877154	TRUE
e13a37ca-e668-4315-b156-f4eeb5814c05	2.814814815	0.01542	0.018804678	TRUE
c3d1dc80-eea8-43f8-8436-5bd0ee6a3b07	inf	0.016427	0.019792102	TRUE
5cb8a505-e571-4faa-ab14-71e7b6a45a89	3.9	0.020267	0.024127924	TRUE

f1f609a2-4fd6-48fc-8a29-3bc39c6ff178	2.887218045	0.022507	0.026124208	TRUE
e2a01ce0-a2fb-435a-9b05-7758e14d4157	2.798611111	0.02232	0.026124208	TRUE
c904090b-149e-4146-9769-f0d2ff965da9	2.571428571	0.022728	0.026124208	TRUE
ceb8fbdf-dd36-4273-b17c-0ee2eca493c5	2.892156863	0.04779	0.05430684	FALSE
c3f677f7-168a-414b-8006-a9cec7da0994	2.26625387	0.061115	0.068668247	FALSE
681955cd-356d-4e64-924b-b0381e3c12bc	4.391891892	0.075823	0.084247489	FALSE
de55dea1-658a-4a84-ae5c-fc554e9c4213	2.433333333	0.147408	0.161987064	FALSE
ca0a0a82-4e82-4559-9666-d19faa3edf8f	3.12	0.174904	0.190113072	FALSE
42959bfe-1a62-465f-aafc-684d973b43f7	5.310344828	0.19418	0.20879578	FALSE
29f824b7-d899-40c1-b53b-edcb3f8e3d2e	4.838709677	0.217135	0.230994584	FALSE
c153c4d8-2005-440d-9249-e8b8f2f73a96	3.578947368	0.303429	0.319398628	FALSE
1ede5c59-96ae-486b-ae1f-5f7c7909cf6a	inf	0.394495	0.410932722	FALSE
8b3a593a-2359-476a-a501-e1baaa4873bb	2.566666667	0.489806	0.504954969	FALSE
2fcb9b43-8a11-4730-b57e-4e9fe9013f1c	2.242424242	0.528542	0.539328881	FALSE
9c10ea63-f990-4c9b-9149-df9583e59610	1.609756098	0.624363	0.630669591	FALSE
95e82e2f-d87e-4785-9e8e-c6b41d8b73f0	0.704545455	0.802657	0.802656636	FALSE
