# Supplementary material for: In-silico personalized protein–protein interaction networks prioritize candidate compounds for glioblastoma
Source: PLoS Comput Biol. 2026 Sep 22;22(9):e1014785. doi: 10.1371/journal.pcbi.1014785 (PMC13596792; doi:10.1371/journal.pcbi.1014785)
Supplement: Supplementary file 3 [file pcbi.1014785.s016.pdf]

Fisher test results for recurrent tumors

Sample	Odds ratio	p value
ddd4c4a7-6057-4244-aa4d-ccffe6e1c95a	10.8	5.5289E-08
d44d370d-d86e-4006-9530-ab3442cb2848	7.733333333	3.49653E-06
f8e7cbd2-b54a-4f30-8fc1-a5c80bfad8b6	6.547619048	1.22401E-05
b4919075-90a9-4c8a-82a0-a0b5f9944ad3	6.610576923	1.15679E-05
fe179a60-5e81-48c1-b437-7c38ed910ba8	6.547619048	1.22401E-05
c12052d7-f5ae-4fd5-a36b-ac0ce21179a6	4.932692308	0.000698789
1142d18f-9cd9-41b4-a9f2-975730cb713a	3.897435897	0.001739076
57c3b272-7dcb-4937-83c1-02a3694e701f	3.561904762	0.002198158
8223f42b-434d-4966-a123-3b8a947884bc	3.423611111	0.004654259
0317a370-6a1e-44bd-bfd0-81fd06ec56fb	2.858823529	0.011839543
f0f7d061-ace0-4dbd-90ec-df8f5985a7a2	2.571428571	0.022728061
928160c7-f711-4b23-832c-8c12aea9fe85	1.633333333	0.266552689

Adjusted P-value	Significant
6.63468E-07	TRUE
2.09792E-05	TRUE
2.93762E-05	TRUE
2.93762E-05	TRUE
2.93762E-05	TRUE
0.001397578	TRUE
0.002981273	TRUE
0.003297237	TRUE
0.006205679	TRUE
0.014207452	TRUE
0.024794248	TRUE
0.266552689	FALSE
